# Chemotaxis of ATPase-Powered Nanoparticles up Extra- and Intracellular ATP Gradients

**DOI:** 10.1021/acs.nanolett.5c06514

**Published:** 2026-05-05

**Authors:** Ekta Shandilya, Xiaotian Lu, Ayusman Sen, Peter J. Butler

**Affiliations:** †Department of Biomedical Engineering, ‡Department of Chemistry, §Department of Chemical Engineering, and ∥Department of Materials Science & Engineering, The Pennsylvania State University, University Park, Pennsylvania 16802, United States

**Keywords:** Chemotaxis, ATPase, Cellular Gradients, Nanoparticles, Liposomes, Mitochondria, Endothelial Cells, HeLa Cells, Enzyme−Substrate Interactions

## Abstract

Guiding synthetic nanomaterials toward specific cells and subcellular organelles remains a critical challenge for targeted therapeutics. Here, we report that ATPase-functionalized nanoparticles harness enzymatic turnover to autonomously navigate extracellular and intracellular ATP gradients, accumulating near cell surfaces, experiencing enhanced uptake, and once endocytosed, localizing selectively to mitochondria in both primary human aortic endothelial cells and HeLa cells. ATP depletion or ATPase inhibition abolishes accumulation and disrupts mitochondrial targeting, confirming the requirement for active enzymatic turnover. This targeting mechanism is preserved across particle types, including lipid-based vesicles, indicating broad applicability. This work establishes enzyme-powered chemotaxis as a route to pericellular accumulation, enhanced endocytosis, and organelle-specific delivery, providing a foundation for responsive nanomedicines targeting metabolically active disease environments. The strategy shifts the paradigm from passive, receptor-based delivery to dynamic, energy-responsive targeting.

## Graphical abstract

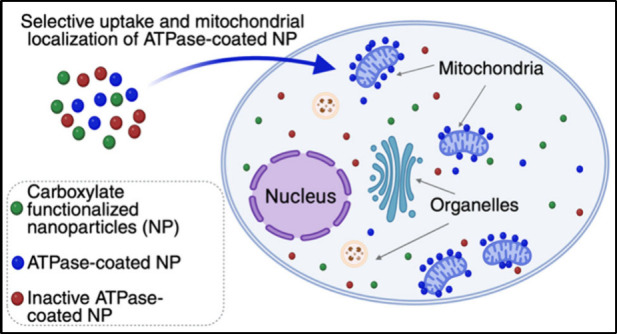

Targeted delivery to specific cells and intracellular organelles remains one of the central challenges in nanomedicine, particularly within the complex and dynamic architecture of living cells.^1,2^ While surface-functionalized nanoparticles (NPs) can achieve selective binding and uptake through ligand–receptor interactions, there are currently no means by which to enhance accumulation of NPs near target cells, thereby enhancing their endocytosis, and there are no mechanisms by which NPs can navigate intracellular compartments with spatiotemporal precision.^3–6^ The targeting of mitochondria is particularly important, since their biochemical landscape and localization are tightly coupled to cellular metabolic states.^7^ Mitochondria play a central role in ATP production,^8^ apoptosis,^9^ calcium signaling,^10^ and redox homeostasis,^11,12^ and are increasingly recognized as critical players in the pathology of diseases such as cancer, cardiovascular dysfunction, type 2 diabetes, and neurodegeneration.^13–15^

In recent years, the field of synthetic active matter has opened new frontiers in nanoscale transport, with enzyme-powered nanomotors demonstrating directional motion in response to substrate gradients in vitro and in complex soft and vesicular systems.^16–23^ The chemotaxis of enzyme-functionalized nanoparticles and vesicles up substrate gradients have been demonstrated for hexokinase, urease, ATPase and glucose oxidase systems under externally imposed gradients.^24–28^ However, translating these principles into live-cell contexts, and leveraging them to achieve subcellular targeting, has not been demonstrated.

ATP, the universal energy currency of the cell, offers a unique opportunity in this context.^29^ It is present in elevated concentrations near mitochondria and membrane-bound ATP synthases,^30^ and is dynamically regulated in response to nutrient availability and cellular stress.^31^ ATP release outside cells occurs through vesicular exocytosis and channel-mediated mechanisms, enabling cells to establish extracellular ATP microenvironments that respond dynamically to mechanical and metabolic cues.^32^ Computational modeling and experiments have shown that such release can sustain steep extracellular ATP gradients within 10s of microns from the cell surface, while reaction-diffusion models predict that intracellular ATP heterogeneity can arise when mitochondria (sources) and ATP-using organelles (sinks) are spatially clustered.^33–35^ These properties make ATP an attractive biochemical signal for real-time navigation. We hypothesized that nanoparticles functionalized with ATP-hydrolyzing enzymes could harness ATP gradients to achieve autonomous transport toward ATP secreting cells and intracellular localization near metabolically active mitochondria.^36,37^

Here, we report the design and implementation of catalytically active nanoparticles functionalized with adenosine triphosphatase from porcine cerebral cortex (NP-A) that exhibit ATP-driven chemotaxis within both primary and cancer cells. Using a combination of fluorescence correlation spectroscopy (FCS), confocal microscopy, and structured illumination microscopy (SIM), we demonstrate that NP-A first accumulates near cell surfaces and then colocalizes with mitochondria in an ATP-dependent manner. Perturbation of ATP levels via nutrient starvation, ATP synthase inhibition, or ATPase inhibition, abolishes this localization, confirming that enzymatic turnover and ATP gradients are essential for enhanced accumulation, uptake, and mitochondrial targeting. Importantly, this mechanism is active across both primary human aortic endothelial cells (HAECs) and immortalized (HeLa) cell lines suggesting broad applicability across cell lines, and is independent of particle composition, as ATPase-coated liposomes exhibit similar behavior.

Our findings establish a new paradigm in organelle-specific delivery wherein enzymatic activity is functionally coupled to cellular metabolism, enabling dynamic and autonomous chemotactic margination toward cells and subcellular targeting. This work expands the scope of activity-dependent intracellular localization and highlights how bioenergetic gradients can be exploited to guide nanocarriers within living systems.

## Design, surface functionalization, and physicochemical characterization of ATPase-coated nanoparticles (NP-A)

Precise cellular targeting remains one of the grand challenges in nanomedicine. Conventional strategies primarily rely on surface ligands for receptor-mediated uptake, which often lack specificity for dynamic organelle states and are insensitive to real-time metabolic activity.^38,39^ Given the central role of mitochondria in regulating ATP production,^30^ reactive oxygen species,^40^ and apoptosis,^9,41^ we designed a system that can autonomously navigate toward ATP-producing cells and, once endocytosed, move toward mitochondria by sensing and responding to cellular ATP concentration gradients. To achieve this, we designed nanoparticles functionalized with porcine cerebral ATPase, aiming to leverage enzymatic turnover of ATP for active chemotactic propulsion.^42,43^ ATP is not only the universal energy currency of the cell but is also spatially concentrated near mitochondria, making it an ideal cue for bioenergetically driven targeting. Nanoparticles (NPs) with carboxylate functionalized fluorescent polystyrene core (100 nm diameter) were conjugated with active hydrolase ATPase (NP-A) using EDC/NHS coupling (Figure 1, details in Supporting Information, SI). The ATPase on NP-A was thermally denatured to form heat-inactivated ATPase coated particles (NP-I) as a negative control (Figure 1).

**Figure 1:**
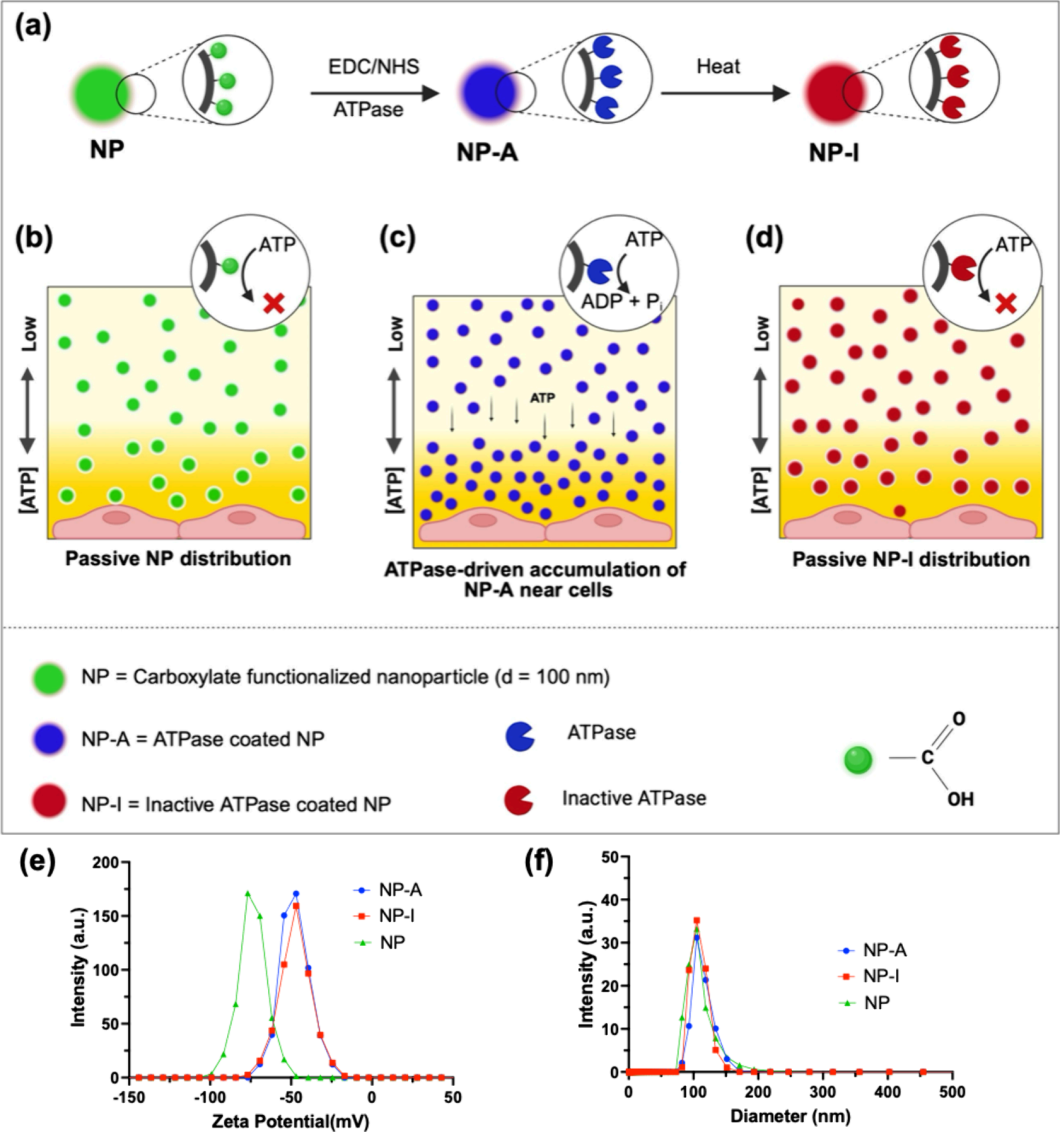
Design, surface functionalization, and physicochemical characterization of ATPase-coated nanoparticles (NP-A). (a) Schematic of nanoparticle modification. Carboxylate-functionalized nanoparticles (NP, green) were conjugated with ATPase via EDC/NHS chemistry to generate catalytically active particles (NP-A, blue). Heat-denatured ATPase was used to create inactive control particles (NP-I, red). (b–d) Conceptual diagrams illustrating particle distributions under ATP gradients. In panels b and d respectively, NP and NP-I distribute uniformly in the medium, while in panel c NP-A actively accumulates near ATP-rich cell surfaces through ATP hydrolysis. (e) ζ potential measurements of NP, NP-A, and NP-I showing conjugation of ATPase results in a clear shift in surface potential relative to bare NP, confirming successful enzyme attachment in 1× PBS at 25 °C. The similar ζ potentials of NP-A and NP-I indicate that heat treatment inactivates ATPase without disrupting its surface association. (f) Dynamic light scattering (DLS) measurements of hydrodynamic diameter for NP, NP-A, and NP-I in 1× PBS at 25 °C. All particle formulations exhibit comparable size distributions centered near 100 nm, indicating that ATPase conjugation and subsequent heat inactivation do not induce aggregation or significant changes in particle size.

To calculate the extent of ATPase conjugation, we quantified unbound ATPase in the supernatant after EDC/NHS coupling using UV absorbance (details in SI). By subtracting the free ATPase from the initial amount, we estimated the number of enzyme molecules per particle and the percentage of theoretical monolayer coverage. Based on this depletion analysis, we calculated an average ATPase surface coverage of 130%, corresponding to bound ATPase concentration of 70 μg/mL and particle density of 3.6 × 10^11^ particles/mL (Figures S1 and S2 and Table S1). Full calculation parameters are provided in the SI.

The surface modification was validated by zeta potential shift. Specifically, the zeta potential of unmodified nanoparticles (NP) was −72.1 ± 8.2 mV, which shifted to −48.2 ± 5.5 mV upon conjugation with ATPase (NP-A) indicating successful surface functionalization. Similarly, NP-I exhibited a ζ potential of −50.7  ±  6.9 mV, indicating that ATPase remained surface-bound even after heat denaturation (Figures 1e and S3). We also measured the size of the nanoparticle before and after the ATPase coating using dynamic light scattering (DLS); the hydrodynamic diameter of the nanoparticles remained close to 100 nm (Figures 1f and S4).

To ensure enzymatic activity was preserved postconjugation, we evaluated ATP hydrolysis kinetics using a luciferase-based assay calibrated with ATP standards (Figure S5, details in the SI).^44^ Michaelis–Menten analysis revealed that NP-A retained substantial catalytic activity with a *V*_max_ of ∼0.4 μM/s and *K*_m_ of ∼15 μM (Figures S5 and S6 and Table S2). In contrast, NP-I is essentially inactive, confirming thermal inactivation of the enzyme without detachment from the nanoparticle surface as confirmed by the zeta potential measurements (Figures 1e and S6).

We should note that chemotactic motion does not necessarily require asymmetric distribution of catalytic centers on a particle.^23,45^ The asymmetry in the substrate gradient dictates the direction of motion. Additionally, several studies have shown that enzymes are unevenly distributed when coated on a variety of hard and soft particles.^46,47^

## Experimental and Computational Analysis of ATPase-Mediated Chemotaxis and Pericellular Accumulation

Based on this characterization, we hypothesized that NP-A could exhibit chemotaxis-like behavior in live-cell environments by actively responding to endogenous ATP gradients. Prior in vitro studies have shown that enzyme-powered nanomotors can migrate along imposed substrate gradients; however, whether this principle could be extended to the complex and dynamic landscape of living cells has remained unknown. In mammalian systems, ATP is not uniformly distributed, it is secreted at the plasma membrane and highly concentrated near mitochondria, generating local gradients that could potentially guide enzymatic nanoparticle accumulation. We reasoned that NP-A might exploit these gradients to navigate toward metabolically active regions, particularly the perimitochondrial space, enabling energy-responsive subcellular targeting.

To recreate physiologically relevant ATP gradients, we cultured human aortic endothelial cells (HAECs) on fibronectin-coated surfaces which promoted robust adhesion and sustained ATP release into the extracellular space. This setup mimics the metabolic activity of vascular endothelium, where ATP is actively secreted and subsequently degraded by membrane-bound ectonucleotidases, resulting in a steep local gradient that decays with increasing distance from the cell surface.

To test whether NP-A responds to these gradients, we performed fluorescence correlation spectroscopy (FCS) measurements at two axial heights above the cells: 5 μm and 20 μm. For each experiment, the apical plasma membrane was first brought into focus using DIC microscopy and defined as *z* = 0 μm; the focal plane was then shifted upward by +5 μm or +20 μm, and FCS data were collected at that fixed position. A schematic overview of the FCS measurement geometry, including both lateral positions and axial heights, is provided in Figure S7a. The 5 and 20 μm measurements were conducted in separate experiments using independent cell preparations to ensure synchronized data collection time points, and to avoid photobleaching or mechanical disturbances from repeated focal plane adjustments on the same cells. The 5 μm height probes the pericellular region with higher ATP concentration due to secretion and mitochondrial proximity, while 20 μm serves as a control region further from the cell where ATP would be diluted and homogenized by diffusion. Because of the resulting steep ATP concentration gradient between 5 and 20 μm, we hypothesized that if NP-A exhibits ATP-guided chemotactic behavior, preferential accumulation would occur near the 5 μm region. This spatial analysis enables direct evaluation of enzyme-powered nanoparticle positioning within biologically relevant ATP gradients generated by living cells. Indeed, under nutrient-rich conditions, we observed that NP-A particles exhibited a ∼2.5-fold higher concentration at 5 μm compared to 20 μm (Figure 2a,b), strongly suggesting ATP-responsive localization near the cell surface. Statistical analysis further supported this trend, with NP-A showing significant separation from NP and NP-I at 5 μm beginning at early time points, while no comparable significance was observed at 20 μm. In contrast, both control particles NP and NP-I showed no significant difference between the two heights (Figure 2a,b), indicating that the spatial accumulation was not due to nonspecific binding, charge effects, or sedimentation. To further confirm that this behavior is driven by ATP gradients produced by live cells, we repeated the same FCS measurements in a cell-free system (Figure S7b,c). In this case, the concentration remained uniform at both 5 μm and 20 μm for all particles (NP, NP-A, and NP-I), again eliminating the possibility that sedimentation due to gravity or surface interactions were responsible for the observed enrichment in live-cell conditions.

**Figure 2:**
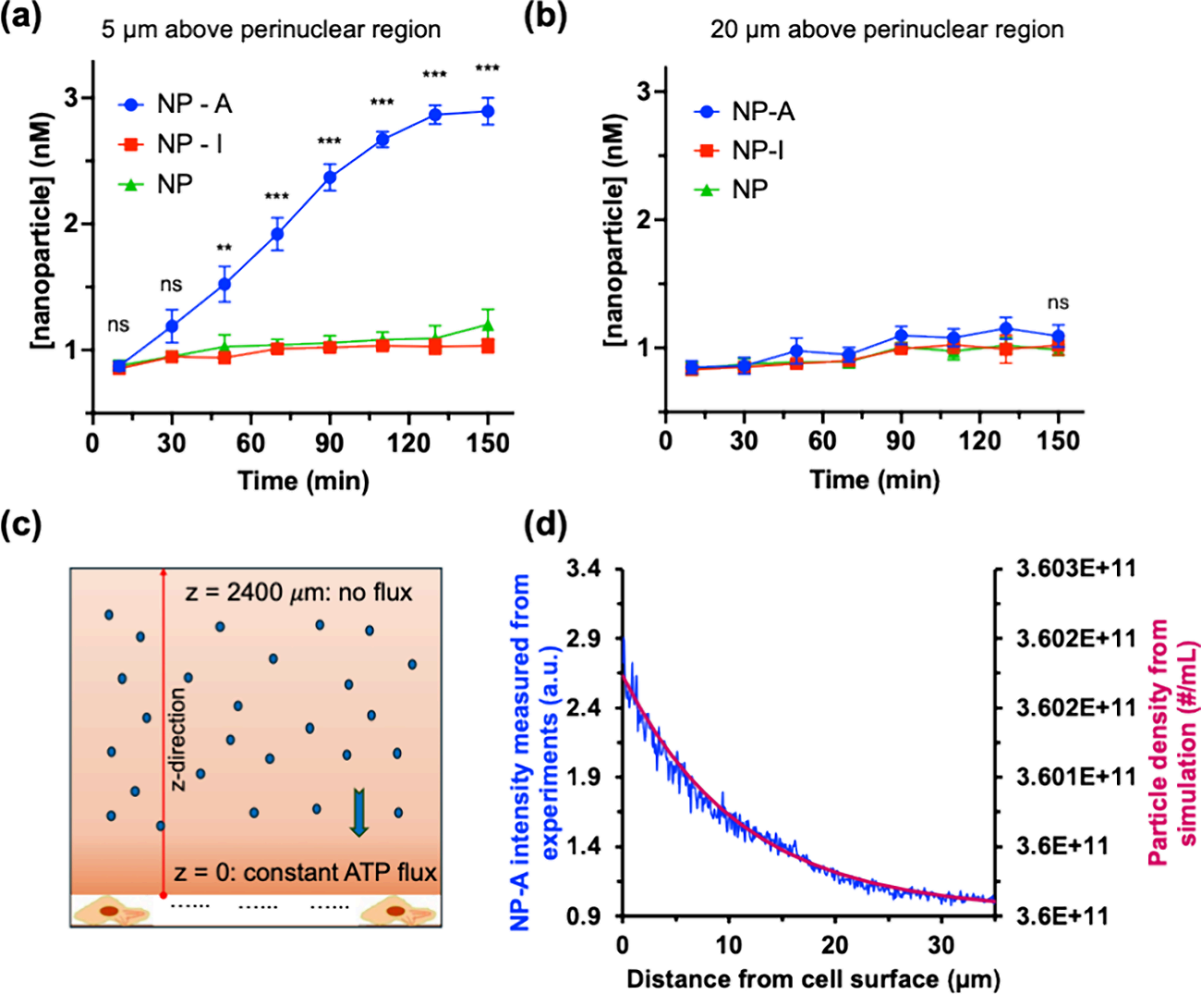
Experimental and computational analysis of ATPase-mediated chemotaxis and pericellular accumulation. (a) Time-dependent nanoparticle concentration measured by fluorescence correlation spectroscopy (FCS) at 5 μm above the perinuclear region of HAECs. NP-A (blue) exhibits a pronounced increase in local concentration over time, whereas NP (green) and NP-I (red) show minimal changes. (b) Corresponding FCS measurements acquired at 20 μm above the cell surface. At this distance from the cells, NP, NP-A, and NP-I display comparable and nearly constant concentrations over time, indicating the absence of particle accumulation at this height. Statistical significance was determined by two-way ANOVA with time and particle type as factors, followed by Šídák’s multiple-comparisons test comparing NP-A with NP-I and NP at each time point; ns, not significant; **p < 0.01, and ***p < 0.001. (c) Schematic of the one-dimensional reaction-diffusion model used to simulate ATP release, gradient formation, and particle transport along the height of the culture medium. Cells at the bottom boundary (*z* = 0 μm) are modeled as a constant ATP flux source, while the top boundary (*z* = 2400 μm) is treated as a no-flux condition. Particle transport includes both diffusion and ATP-gradient-driven chemotactic drift. (d) Representative particle fluorescence intensity profile (blue solid trace) extracted from structured illumination microscopy (SIM) *z*-stack imaging after 2 h of incubation using ImageJ, showing enhanced signal near the cell surface with a decrease in intensity with increasing distance from the cell surface. The red curve shows the corresponding simulated particle density profile as a function of distance from the cell surface.

Additionally, we extended our FCS analysis to the cell periphery, distinct from the perinuclear region analyzed in Figure 2, a region where mitochondria are scarce but extracellular ATP is still released by cells. In this peripheral zone, NP-A exhibited a time-dependent increase in local concentration at 5 μm, that was less than in the perinuclear region, while NP-I and NP time-dependent concentrations remained flat over time, consistent with gradient-driven chemotactic accumulation near the cell (Figure S7d). In contrast, no significant enrichment was detected at 20 μm or under cell-free conditions (Figure S7e), confirming that active accumulation toward membrane-released ATP operates independently of direct mitochondrial association. These observations confirmed that the accumulation of NP-A near the cell surface occurs only when there are ATP gradients generated by cells, suggesting that ATPase-mediated chemotaxis enables particles to autonomously localize near active cell surfaces. The above findings were further supported by structured illumination microscopy (SIM) z-stack imaging while slicing the frames going in upward direction from the cell surface, and this experiment further revealed enhanced axial accumulation of NP-A near the cell surface, in contrast to NP and NP-I, which exhibited diffuse and low-intensity distributions throughout the imaging depth (Figure 2d versus Figure S8a).

We also developed a one-dimensional reaction-diffusion model to assess whether ATP release from endothelial cells can generate spatial gradients sufficient to drive ATPase-mediated particle accumulation (Figure 2c, details in the SI). Using experimentally measured ATP release rates, ATPase kinetics, particle diffusion coefficients, and particle number densities, we simulated ATP diffusion and consumption along the height of the culture medium above the cells. The model predicts the formation of a steep ATP gradient confined to the pericellular region, with rapid decay toward bulk concentrations over micrometer length scales (Figure S8b). When chemotactic drift driven by ATP gradients is incorporated into the particle transport equation, the simulation yields enhanced particle accumulation near the cell surface followed by a gradual relaxation to uniform particle density at larger distances (Figure 2d). The simulated particle distribution profile has a similar characteristic decay length to that of the axial NP-A intensity profile observed in experiments. To further distinguish the role of enzymatic activity in driving particle accumulation, we also simulated the behavior of NP-I particles. In this case, ATP consumption and chemotactic drift were omitted from the model, and particle transport was governed solely by diffusion. Under these conditions, the simulation resulted in no accumulation of particles near the cell surface and a nearly uniform particle distribution throughout the height of the medium. This behavior contrasts sharply with the ATPase-active case and is consistent with experimental observations, where NP-I shows no near-surface enrichment in z-stack imaging or FCS measurements (Figures 2 and S7 and S8). Together, these results confirm that particle accumulation near the cell surface requires ATPase activity and cannot be explained by passive diffusion alone. These results also support the conclusion that ATPase activity at the nanoparticle surface enables particles to sense and respond to cellular ATP gradients. This behavior represents active chemotactic migration toward metabolically active zones by harnessing energy-rich chemical cues. Importantly, these results establish a broader design principle: physiologically relevant gradients, even those extending only a few microns, can be effectively exploited for cellular targeting, provided the enzymatic propulsion system is appropriately tuned to the biochemical environment of the cell.

## Active ATPase-Functionalized Nanoparticles Exhibiting Preferential Mitochondrial Localization

Building on these observations, we next asked whether the pericellular accumulation of NP-A could bias intracellular localization toward specific organelles. Given that mitochondria represent major intracellular sources of ATP, we hypothesized that catalytically active nanoparticles would preferentially localize near mitochondria following cellular entry. To test this, we labeled mitochondria using MitoTracker Deep Red, a dye that accumulates in active mitochondria based on membrane potential, enabling us to evaluate whether intracellularly localized particles exhibit organelle-specific targeting. Indeed, NP-A particles showed strong colocalization with mitochondria (Figure 3a), supporting organelle-specific targeting. In contrast, control particles (NP and NP-I) displayed diffuse cytoplasmic distribution or minimal internalization with no evidence of mitochondrial association (Figures 3b and S9). To quantitatively assess subcellular targeting, we performed Pearson’s correlation analysis to evaluate the degree of colocalization between nanoparticles and mitochondria across all experimental conditions. Under nutrient-rich conditions, NP-A showed a strong correlation with mitochondrial signal (*r* = 0.55  ±  0.07), whereas NP-I exhibited minimal overlap (*r* = 0.19 ±  0.02), consistent with the role of enzymatic activity in driving organelle-specific targeting. We also observed that cells incubated with NP-A exhibited markedly higher intracellular fluorescence compared to NP and NP-I controls (Figure 3c) measured using ImageJ, with punctate fluorescence patterns indicating higher internalization with active ATPase after 2 h of incubation.

**Figure 3:**
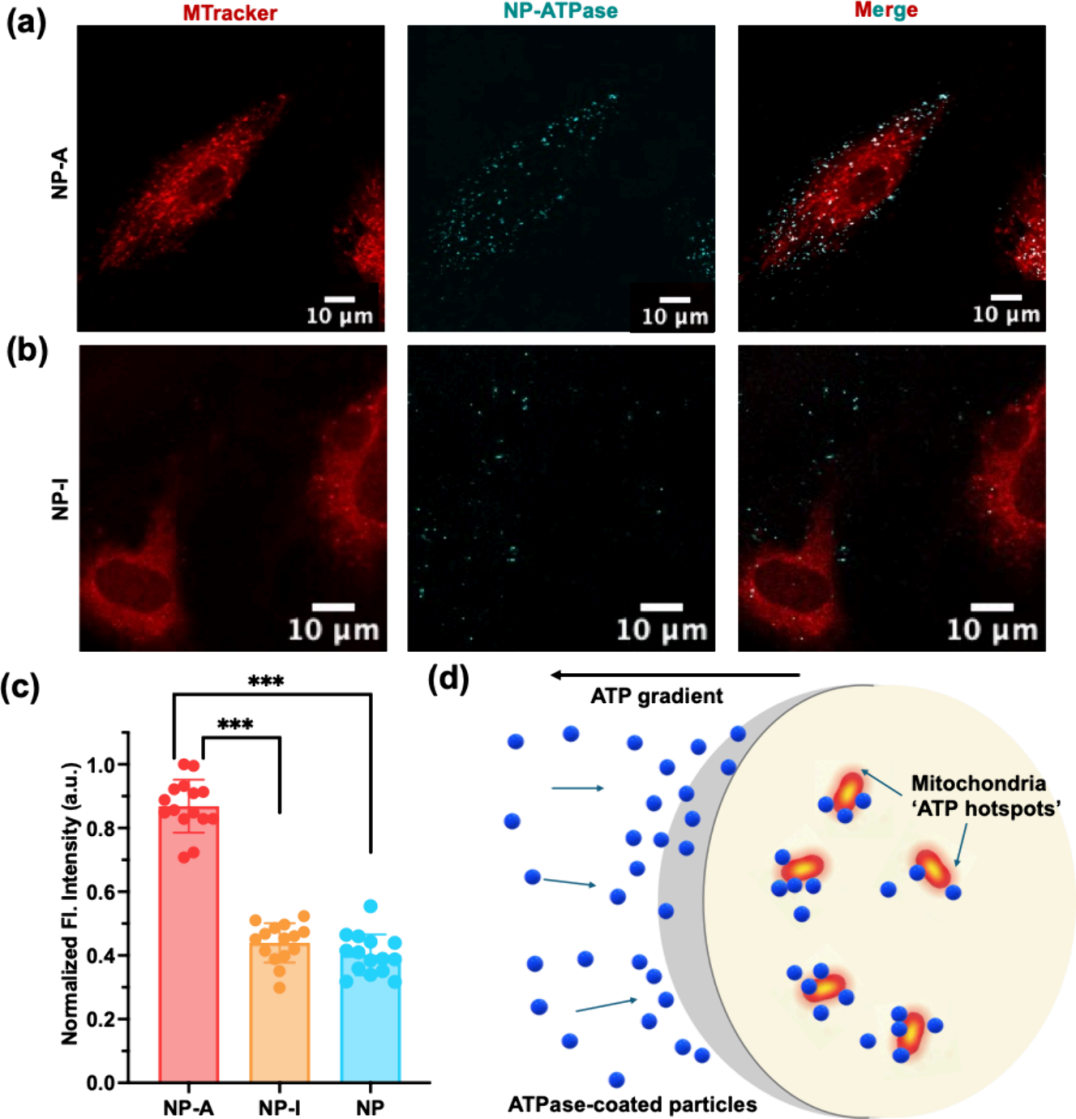
Active ATPase-functionalized nanoparticles exhibit preferential mitochondrial localization. (a) Confocal fluorescence images of HAECs incubated with ATPase-coated nanoparticles (NP-A). Mitochondria are labeled with MitoTracker (red), nanoparticles are shown in cyan, and the merged image highlights pronounced colocalization of NP-A with mitochondria. Scale bars, 10 μm. (b) Corresponding confocal images of cells incubated with inactive ATPase-coated nanoparticles (NP-I). NP-I exhibits reduced intracellular signal and minimal colocalization with mitochondria under identical conditions. Scale bars, 10 μm. (c) Quantification of normalized intracellular fluorescence intensity for NP-A, NP-I, and bare nanoparticles (NP), showing significantly higher accumulation of NP-A compared to both control particles. Each data point represents an individual cell; bars indicate mean ± s.d. Statistical significance between NP-A and NP-I/NP was assessed using a two-sided Student’s *t* test (*p* < 0.001 (***)). (d) Schematic illustration showing ATPase-mediated intracellular localization. ATPase-coated nanoparticles accumulate preferentially in regions of elevated ATP concentration, with mitochondria acting as intracellular ATP-rich hotspots. This activity-dependent bias leads to enhanced localization of NP-A near mitochondria following cellular uptake.

### Ruling Out Lysosomal Entrapment

To directly test whether mitochondrial localization reflects lysosomal escape followed by passive redistribution, we performed co-staining with LysoTracker Red under identical incubation conditions (Figure S10). NP-A showed minimal colocalization with lysosomes (Pearson’s *r* = 0.07), in sharp contrast to its strong colocalization with mitochondria (*r* = 0.55 ± 0.07; Table S4). This suggests that the preferential mitochondrial association reflects active intracellular redistribution rather than default vesicular trafficking.

### Ruling Out Passive Diffusion

NP-A and NP-I are physicochemically near-identical, carrying the same polystyrene core, identical surface coating density, and statistically indistinguishable zeta potentials (NP-A, −48.2 ± 5.5 mV; NP-I, −50.7 ± 6.9 mV; Figures 1e and S3). The only difference between them is catalytic activity. Despite internalizing under identical conditions, NP-I shows minimal mitochondrial colocalization (*r* = 0.19 ± 0.02) compared to NP-A (*r* = 0.55 ± 0.07; Table S4). Since any passive process would act equally on both particle types given their identical surface properties, passive diffusion cannot account for the selective localization of NP-A.

### Ruling Out Direct ATPase-Mitochondrial Membrane interaction

NP-A does not carry known mitochondrial targeting moieties such as lipophilic cations or targeting peptides, and porcine cerebral ATPase is not known to bind mitochondrial outer membrane proteins. Receptor-mediated or electrostatic docking to the mitochondrial membrane is therefore unlikely. TMRM measurements further confirm no significant change in mitochondrial membrane potential upon NP-A treatment (Figure S11), ruling out electrostatic attraction as a driver of localization.

### Positive Evidence for ATP-Gradient-Driven Chemotaxis

Mitochondrial colocalization of NP-A decreases progressively under glucose starvation (*r* = 0.35 ± 0.03) and oligomycin treatment (*r* = 0.15 ± 0.02), and is nearly abolished under orthovanadate treatment (*r* = 0.18 ± 0.02; Table S4). The orthovanadate result is particularly informative: it inhibits surface-bound ATPase without depleting cellular ATP, yet targeting is lost. This confirms that active enzymatic turnover at the nanoparticle surface is indispensable, consistent with the phoretic chemotaxis mechanism wherein directed particle transport scales with local substrate gradient.

Taken together, the LysoTracker data combined with the NP-A vs NP-I comparison and the orthovanadate result collectively support a model of ATP-gradient-driven chemotactic redistribution within the cytosol following internalization, rather than passive diffusion after lysosomal escape or direct membrane interaction.

Thus, our results support multiple functional roles for enzymatic activity: (i) mediating directional accumulation near ATP-releasing membranes via catalytic propulsion, (ii) enhancing intracellular uptake, and (iii) preferential trafficking to mitochondria. The dependence of both uptake and mitochondrial targeting on catalytic turnover underscores the unique potential of metabolic gradients, particularly ATP distribution, as navigational cues for intracellular nanocarrier delivery. This establishes a foundation for energy-responsive targeting platforms capable of autonomous localization to bioenergetically active subcellular compartments. A schematic summary of this behavior is shown in Figure 3d, illustrating how ATPase-functionalized nanoparticles preferentially localize near intracellular ATP-rich regions, with mitochondria acting as dominant ATP hotspots following cellular uptake.

## Metabolic Perturbations Disrupting Mitochondrial Localization of ATPase-Functionalized Particles (NP-A) without Inducing Cytotoxicity

To further assess whether the targeting behavior was indeed driven by metabolic cues rather than nonspecific interactions or passive sedimentation, we next probed the system’s sensitivity to bioenergetic disruption. Three orthogonal perturbation strategies were employed: (1) glucose starvation to reduce intracellular ATP supply,^48,49^ (2) oligomycin treatment (OA) to block ATP synthesis via inhibition of mitochondrial ATP synthase,^50,51^ and (3) sodium orthovanadate treatment (NV) to directly inhibit ATPase activity.^52^ These conditions allowed us to decouple the contributions of ATP gradient strength and enzymatic propulsion to nanoparticle targeting behavior.

To verify that our bioenergetic perturbations successfully modulated ATP availability, we first measured extracellular ATP concentrations using a luciferase-based bioluminescence assay calibrated with ATP standards (Figure S5).^53,54^ ATP levels were normalized to total cellular protein content determined by a BCA assay to account for variations in cell number across conditions (Figure S12). As expected, both nutrient starvation and oligomycin treatment significantly reduced ATP levels relative to nutrient-rich controls, while sodium orthovanadate, an ATPase inhibitor did not deplete bulk ATP but blocked its enzymatic turnover (Figures 4a and S13). These results confirm that each perturbation selectively modulates different aspects of cellular energy metabolism: substrate availability, ATP synthesis, and ATPase-driven hydrolysis.

**Figure 4:**
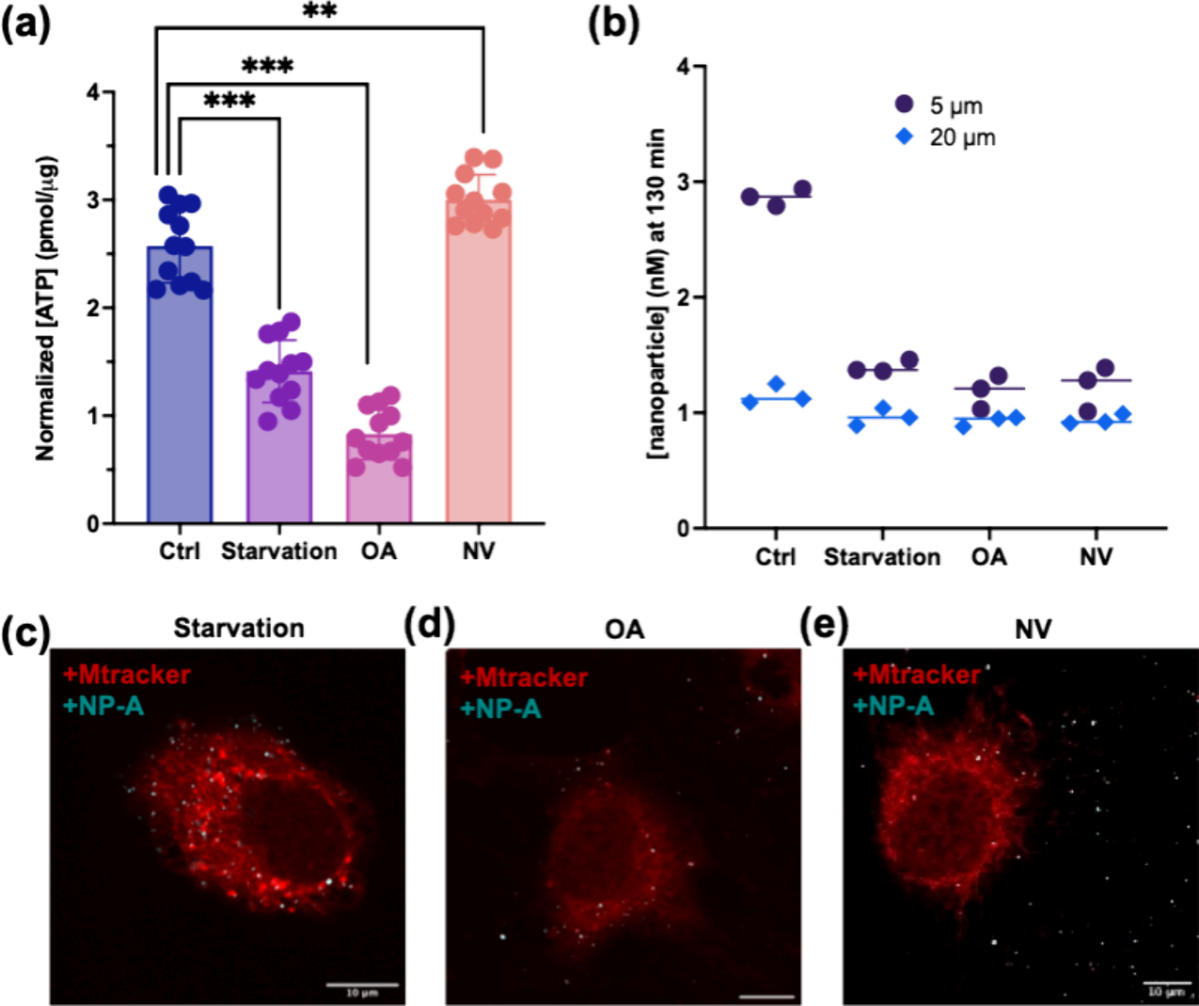
ATP depletion abolishes NP-A accumulation and mitochondrial targeting. (a) Quantification of extracellular ATP levels using a luciferase-based assay confirms significant ATP reduction under starvation, oligomycin, and sodium orthovanadate (NV, Na_3_VO_4_) treatment in HAECs. Statistical significance between control and treated cells was assessed using a two-sided Student’s *t* test (*p* < 0.001 (***); *p* < 0.01 (**)). (b) Median NP-A concentration measured by FCS at 5 and 20 μm above the cell surface. Under nutrient-rich conditions, particles accumulate near the cell surface (5 μm); this gradient disappears under ATP-disrupting conditions, where NP-A distribution is uniform across heights. (c, d) Colocalization of NP-A (cyan) with mitochondria (MitoTracker, red) under nutrient-rich, starvation and oligomycin conditions showing decreased uptake and diminished—but not abolished—targeting. (e) Minimal NP-A colocalization in Na_3_VO_4_-treated cells, indicating severe impairment of mitochondrial targeting under ATPase inhibition. Scale bars = 10 μm. The normalization protocol is described in the SI.

To assess the impact of these metabolic changes on nanoparticle localization behavior, we performed FCS measurements at 5 μm and 20 μm above the cell surface under each condition. In nutrient-rich environments (Figure 4b), NP-A exhibited a strong height-dependent enrichment, with significantly higher particle concentration at 5 μm compared to 20 μm consistent with chemotactic accumulation near ATP-rich pericellular zones. However, this NP concentration gradient was sharply attenuated under all perturbation conditions (Figures 4b and S14 and S15), resulting in uniform NP-A distribution across both heights. These findings indicate that localized ATP production and active enzymatic turnover are *both* essential for driving particle accumulation. Notably, the loss of spatial enrichment in sodium orthovanadate-treated cells underscores that it is not merely the presence of ATP, but the ability of surface-bound ATPase to catalyze its hydrolysis, which enables directed particle localization. Again, consistent with our FCS data, using confocal imaging, we observed that under glucose starvation and oligomycin treatment, both NP-A uptake and mitochondrial colocalization were moderately reduced compared to nutrient-rich conditions, indicating that ATP depletion weakens, but does not completely abolish the ATP gradient-driven accumulation (Figure 4c). Also, upon glucose starvation or oligomycin treatment, NP-A colocalization decreased moderately (*r* = 0.35 ±  0.03 and *r* = 0.15 ±  0.02, respectively), while sodium orthovanadate treatment resulted in further loss of targeting (*r* = 0.18 ±  0.02), approaching the baseline observed for NP-I. These results confirm that mitochondrial localization is both activity-dependent and sensitive to cellular energy status (Table S4). In contrast, sodium orthovanadate treatment nearly eliminated mitochondrial localization, highlighting that enzymatic activity at the nanoparticle surface is essential for directional navigation, not just the presence of ATP. These results establish that ATPase-mediated propulsion and endogenous ATP gradients are both critical for achieving bioenergetically guided subcellular targeting. They also further underscore the broader principle that dynamic metabolic signals can serve as navigational cues for enzyme-powered nanoparticles, enabling autonomous localization to active organelles such as mitochondria.

To determine whether the loss of mitochondrial targeting under energy-disrupting conditions was a genuine consequence of altered bioenergetics, and not due to nanoparticle-induced cytotoxicity or mitochondrial damage, we assessed mitochondrial function and cell viability. Mitochondrial membrane potential, a key indicator of mitochondrial integrity, was measured using TMRM (tetramethylrhodamine methyl ester) staining. The overall cell health was also evaluated via MTT (3-(4,5-dimethylthiazol-2-yl)-2,5-diphenyltetrazolium bromide) viability assays. Across all treatment conditions including nutrient deprivation, oligomycin, and orthovanadate treatment, we assessed mitochondrial membrane potential (TMRM) and cell viability (MTT) both in the presence and absence of NP-A. No significant loss in membrane potential or cell viability was observed under any condition (Figures S11 and S16), confirming that neither the metabolic perturbations themselves nor exposure to NP-A induced cytotoxicity or mitochondrial damage. To further confirm that the observed loss of NP-A targeting under metabolic perturbation was not an artifact of impaired endocytic capacity, we quantified nanoparticle uptake by flow cytometry across all treatment conditions (Figures S17 and S18). Under nutrient-rich conditions, NP-A exhibited a median fluorescence intensity (MFI) approximately 4-fold higher than NP-I (4074 ± 380 vs 1053 ± 114; *p* < 0.001), consistent with chemotaxis-enhanced pericellular accumulation driving increased cellular uptake. Glucose starvation moderately reduced NP-A uptake (MFI = 2091 ± 98; *p* < 0.001 vs NP-I), while oligomycin A and sodium orthovanadate treatment reduced NP-A uptake to levels statistically indistinguishable from NP-I (*p* ≥ 0.05). Importantly, NP-I uptake remained constant across all conditions (MFI range: 1004–1069), demonstrating that the endocytic machinery of the cells was unaffected by any of the metabolic perturbations. These data confirm that the uptake advantage of NP-A is entirely dependent on active chemotactic accumulation near the cell surface, and that when this gradient-driven enrichment is abolished, NP-A behaves identically to a catalytically inactive particle.

Together, these results reinforce a key mechanistic insight: both the spatial cue (ATP gradient) and the catalytic capability (ATPase activity) are essential for directional subcellular targeting. ATP defines a localized energy landscape, and enzymatic turnover allows the nanoparticle to actively sense and respond to that landscape. This requirement distinguishes our system from passive diffusion or receptor-mediated delivery, establishing a novel framework for activity-dependent, energy-responsive intracellular localization.

## ATPase-Mediated Targeting Generalizable across Cell Types and Nanoparticle Architectures

To evaluate the generalizability of ATPase-driven mitochondrial targeting, we first tested whether this mechanism was effective in other mammalian cell types beyond HAECs. HeLa cells were incubated with NP-A and analyzed by confocal microscopy. Similar to our findings in endothelial cells, NP-A demonstrated robust intracellular uptake and strong colocalization with mitochondria in HeLa cells (Figure 5a). These results indicate that ATP-gradient-driven chemotactic behavior is not restricted to a specific cell lineage and instead reflects a broadly applicable mechanism tied to conserved features of mitochondrial metabolism. To quantitatively assess whether metabolic differences between cell types influence targeting efficiency, we measured extracellular ATP release from both HAECs and HeLa cells under nutrient-rich conditions using a luciferase-based bioluminescence assay normalized to total cellular protein. HeLa cells released approximately 2-fold higher extracellular ATP (5.38 ± 0.29 pmol/μg protein) compared to HAECs (2.57 ± 0.34 pmol/μg protein; *p* < 0.001) (Figure S19), consistent with the elevated glycolytic flux characteristic of cancer cell metabolism. Pearson’s correlation analysis revealed that NP-A achieved significantly higher mitochondrial colocalization in HeLa cells (*r* = 0.63 ± 0.05) compared to HAECs (*r* = 0.55 ± 0.07; *p* < 0.01), while NP-I showed consistently low colocalization in both cell types (HeLa, *r* = 0.20 ± 0.03; HAECs, *r* = 0.19 ± 0.02; p = 0.39, not significant) (Table S4). The statistically indistinguishable colocalization of NP-I across both cell types confirms that the enhanced mitochondrial targeting of NP-A is activity-dependent and not attributable to differences in mitochondrial morphology or general perinuclear accumulation between the two cell types. This correlation between higher extracellular ATP and enhanced mitochondrial targeting across cell types mirrors the within-HAEC perturbation data, where conditions that reduce ATP availability progressively diminish NP-A colocalization (Table S4), together indicating that targeting efficiency is correlated with cellular metabolic state.

**Figure 5:**
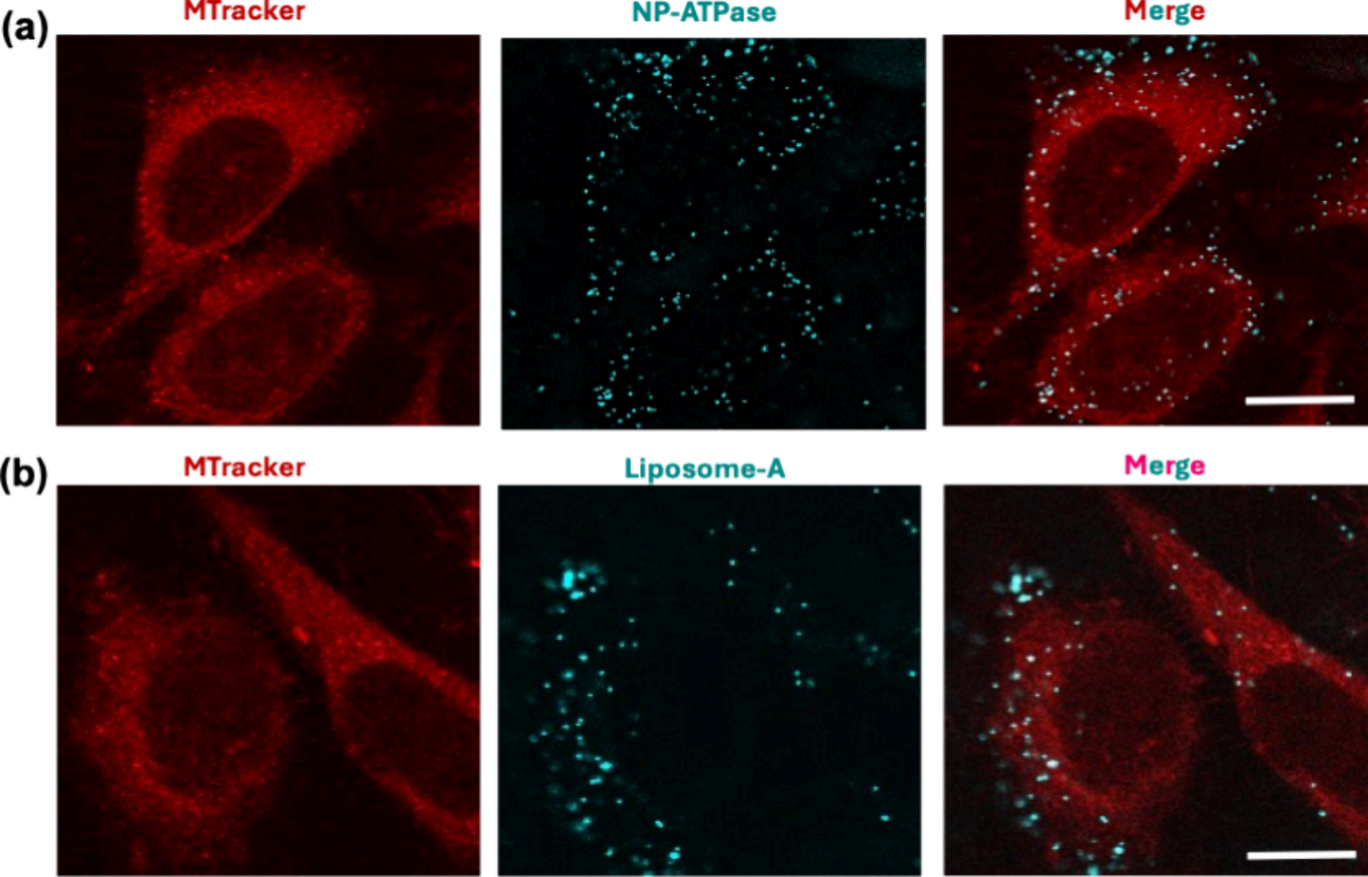
ATPase-driven mitochondrial targeting conserved across particle types and cell lines. (a) Confocal microscopy of HeLa cells stained with MitoTracker (red) and incubated with NP-A (cyan), demonstrating colocalization with mitochondria. (b) Confocal images of HeLa cells treated with ATPase-coated liposomes (liposome-A), also showing mitochondrial colocalization. These results support that ATPase-mediated targeting is effective across cell types and is independent of material properties of the particle material (hard vs soft). Scale bars = 10 μm.

We next asked whether the material properties of the nanoparticle carrier influence targeting efficacy. While our prior experiments employed rigid, polystyrene-based nanoparticles, many clinically relevant drug delivery platforms use soft, deformable systems such as liposomes. To test the compatibility of our strategy with such materials, we synthesized ATPase-functionalized liposomes (liposome-A) via a standard phospholipid formulation and enzyme conjugation protocol (details in SI). After preparing liposomes with and without ATPase we also measured the size of liposomes using DLS, and for both cases the hydrodynamic diameter of the liposomes was around 100 nm (Figure S20). Upon incubation with HAECs, liposome-A particles exhibited significant intracellular uptake and mitochondrial localization, comparable to NP-A behavior (Figure 5b). These findings demonstrate that enzymatic propulsion and ATP gradient sensing are not limited by particle stiffness or composition.

The results described establish that ATPase-mediated subcellular targeting by chemotactic particles is modular, material-agnostic, and cell-type independent. Whether using soft or hard nanoparticles, and in endothelial or HeLa cells, the ATP-responsive system consistently enables autonomous mitochondrial localization. This metabolically guided navigation strategy operates without synthetic ligands, receptors, or external fields, enabling a new class of intelligent, energy-sensing nanocarriers tailored to dynamic cellular states. While NPs can be targeted for cells via ligands for endogenous membrane receptors upregulated in diseased tissue,^1,2,4^ this chemotargeting approach faces a fundamental physical limitation: NPs are small and do not appreciably marginate to the endothelium where therapeutic delivery is needed. Our chemotactic NP-A particles overcome this bottleneck by autonomously accumulating near the cell surface via ATP gradients produced by the cells. This mechanism complements conventional chemotargeting, since NP-A could be further functionalized with receptor-binding ligands, with chemotaxis-driven pericellular enrichment amplifying both passive and receptor-mediated uptake. Crucially, NP-A also targets metabolically active tissue and intracellular mitochondria^7^ through a mechanism that is completely independent of ligand–receptor interactions, a capability that is unavailable in current passive or active targeting strategy, making this platform particularly innovative.

Altogether, these findings define a novel extracellular and intracellular navigation framework where enzymatic activity and endogenous metabolic gradients jointly govern nanoparticle behavior. By leveraging ATP both as fuel and as a spatial cue, this strategy shifts the paradigm from passive, receptor-based delivery to dynamic, energy-responsive targeting. Such a mechanism holds significant potential for future therapeutic development, particularly in disorders marked by mitochondrial dysfunction, metabolic heterogeneity, or altered bioenergetics - such as cancer, ischemia, and neurodegeneration.

This study establishes a generalizable strategy for organelle-specific nanoparticle targeting based on enzymatic propulsion and intracellular energy landscapes. By conjugating ATPase enzymes to nanoparticle surfaces, we harnessed endogenous ATP gradients emanating from mitochondria, as navigational cues for chemotactic accumulation and mitochondrial localization. Our findings reveal that catalytic turnover is essential not only for directional movement but also for enhancing cellular uptake and organelle-specific engagement. These behaviors were conserved across cell types and nanoparticle architectures, demonstrating the modularity and versatility of the platform. Unlike conventional receptor-based or passive delivery methods, this approach leverages dynamic metabolic cues intrinsic to the cellular microenvironment, enabling autonomous, energy-responsive targeting without the need for synthetic ligands or external control. As such, ATPase-functionalized nanoparticles represent a new class of smart nanocarriers that integrate propulsion, sensing, and localization in a single construct, opening avenues for the development of adaptive therapeutic and diagnostic systems capable of responding to cellular metabolic state in real time. Looking forward, this strategy may be adapted to deliver bioactive payloads to metabolically active tissues, offering new opportunities for treating diseases characterized by aberrant energy metabolism.^7,22,38^ The tumor microenvironment represents a particularly compelling application context. Extracellular ATP in tumor interstitial fluid reaches the high micromolar range due to cell turnover, hypoxic stress, and purinergic signaling,^55,56^ and our model framework suggests that such elevated concentrations would further amplify chemotactic driving forces relative to those observed under primary endothelial cell conditions. Beyond oncology, ischemia-reperfusion injury,^7^ where reperfusion triggers acute ATP fluctuations and mitochondrial stress, and neurodegenerative diseases such as Parkinson’s disease and ALS,^13^ where selective mitochondrial dysfunction alters local ATP production, represent additional high-priority contexts in which ATPase-functionalized nanocarriers may enable precise cell-selective intracellular targeting. In principle, the concept of activity-dependent intracellular localization mediated by enzyme-functionalized nanoparticles can be extended beyond ATP gradient-driven mitochondrial targeting, offering a versatile platform for organelle-specific targeting based on enzyme-functionalized hard and soft particles responding to endogenous biochemical gradients.

## Supplementary Material



## Data Availability

The data that supports the findings of this study are presented in Supporting Information. Further information can be obtained from PJB and AS upon reasonable request.

## Notes

Materials; methods: culturing endothelial and HeLa cells; ATPase nanoparticles functionalization; liposome preparation and ATPase functionalization; fluorescence correlation spectroscopy; ATP release simulation; gradient formation; particle chemotaxis; imaging and analysis; flow cytometry; inhibitor treatments and starvation protocol; ATP release measurement and protein normalization; MTT assay; data analysis and normalization; statistical analysis (PDF)
