# Supplementary material for: Chemotaxis of ATPase-Powered Nanoparticles up Extra- and Intracellular ATP Gradients
Source: Nano Lett. 2026 May 5;26(21):6828–38. doi: 10.1021/acs.nanolett.5c06514 (PMC13237814; doi:10.1021/acs.nanolett.5c06514)
Supplement: Supplementary file 1 [file nl5c06514_si_001.pdf]

Supporting Information

Chemotaxis of ATPase-Powered Nanoparticles up Extra-and Intra-Cellular ATP Gradients

Ekta Shandilya,[1,2] Xiaotian Lu,[3] Ayusman Sen,[2,3,4]* and Peter J. Butler[1]*

[1]Department of Biomedical Engineering, [2]Department of Chemistry, [3]Department of Chemical Engineering, and [4]Department of Materials Science & Engineering,

The Pennsylvania State University, University Park, Pennsylvania 16802, USA.

Emails: Ayusman Sen, asen@psu.edu; Peter J. Butler, pbutler@psu.edu

Table of Contents

A. Materials *S2*

B. Methods *S2*

1. **Culturing Endothelial cells** **S2**
2. **Culturing HeLa Cells** **S3**
3. **ATPase Functionalization of Nanoparticles** **S3**
4. **Liposome preparation and ATPase functionalization** **S4**
5. **Fluorescence Correlation Spectroscopy (FCS)** **S5**
6. **Simulation of ATP release, gradient formation, and particle chemotaxis** **S6**
7. **Imaging and Analysis** **S8**
8. **Flow Cytometry** **S9**
9. **Inhibitor Treatments and Starvation Protocol** **S10**
10. **ATP Release Measurement and Protein Normalization** **S10**
11. **MTT assay** **S11**
12. **Data Analysis and Normalization.** **S12**
13. **Statistical analysis.** **S12**

C. Supplementary Figures S1-S20 .... *S13*

D. Supplementary Table S1-S4 .... *S30*

E. References *S33*

A. Materials

The study employed a comprehensive set of materials to investigate ATPase-powered nanoparticle chemotaxis. All chemicals and materials were used as purchased without further purification. Human aortic endothelial cells (HAECs; Lonza, CC-2535) and HeLa cells (ATCC® CCL-2™) were used as biological models, cultured in EBM-2 medium with EGM-2 supplements and EMEM with fetal bovine serum, respectively. For nanoparticle experiments, 100 nm carboxylate-modified fluorescent polystyrene beads (Invitrogen) were functionalized with ATPase enzyme (adenosine triphosphatase from porcine cerebral cortex; Sigma-Aldrich) via EDC (1-ethyl-3-(3-dimethylaminopropyl) carbodiimide hydrochloride; Sigma-Aldrich) and NHS (N-hydroxysuccinimide; Sigma-Aldrich) crosslinking chemistry. Fluorescent labeling of liposomes was performed with Rhodamine PE; Avanti Polar Lipids. Liposomes were prepared from Egg PC (egg phosphatidylcholine; Avanti Polar Lipids), cholesterol (Sigma-Aldrich), and DSPE-PEG2000 (1,2-distearoyl-sn-glycero-3-phosphoethanolamine-N-[methoxy(polyethylene glycol)-2000]; Avanti Polar Lipids) using an extrusion process through polycarbonate membranes. Purification was achieved with ultrafiltration devices (Amicon Ultra, Millipore). For imaging, a Leica fluorescence microscope equipped with a 60× oil-immersion objective was used, alongside organelle-specific dyes such as MitoTracker™ Deep Red FM (Thermo Fisher Scientific). Additional fluorescent calibration utilized RhB (Rhodamine B; Sigma-Aldrich). Flow cytometry was performed with a BD LSR (Becton Dickinson Laser Scanning Research) Fortessa cytometer. Metabolic inhibitors included Oligomycin A (Sigma-Aldrich) and sodium orthovanadate (Thermo Fisher Scientific). Biochemical assays employed the MTT reagent (3-(4,5-Dimethylthiazol-2-yl)-2,5-diphenyltetrazolium bromide; Thermo Fisher Scientific), the BCA (bicinchoninic acid) protein assay kit (Thermo Fisher Scientific), and the luciferin-luciferase ATP Determination Kit (Molecular Probes, Thermo Fisher Scientific). Phosphate-buffered saline (PBS; Corning) was used throughout for washing and buffer preparations.

B. Methods

1. Culturing Endothelial cells

Human aortic endothelial cells (HAECs; Lonza, CC-2535) were cultured in Endothelial Basal Medium (EBM-2) supplemented with EGM-2 BulletKit growth factors (Lonza, CC-3162), according to the manufacturer's instructions. Prior to seeding, tissue culture dishes were coated with fibronectin (50 μg/mL; Sigma-Aldrich) for 1 h at room temperature to promote endothelial adhesion. HAECs were seeded onto the coated surface and allowed to adhere under static conditions for 1 h at 37 °C in a humidified incubator with 5% CO_2. Following attachment, complete growth medium was added gently to the culture dishes, and cells were maintained by replacing the medium every 24 h. All experiments were performed using cells at passages 4–8 to ensure phenotypic consistency.

2. Culturing HeLa Cells

HeLa cells (ATCC® CCL-2™) were obtained from the American Type Culture Collection and maintained according to the supplier's recommendations. Cells were cultured in Eagle's Minimum Essential Medium (EMEM) (ATCC 30-2003) supplemented with 10% fetal bovine serum (FBS) (ATCC 30-2020) and 1% penicillin–streptomycin. Cultures were maintained in a humidified incubator at 37°C with 5% CO_2 and passaged at 70–90% confluence using 0.25% trypsin-EDTA. For all experiments, cells were seeded on fibronectin-coated glass-bottom dishes and allowed to adhere overnight before nanoparticle treatment.

3. ATPase Functionalization of Nanoparticles

For all experiments, 100 nm carboxylate-modified fluorescent particles (orange fluorescence; Thermo Fisher Scientific, F8800) were used. To eliminate residual sodium azide from the storage buffer, 50 µL of bead suspension was centrifuged at 15,000 × g for 10 min and washed twice with sterile Milli-Q water. The washed bead suspension was diluted to a final volume of 6 mL and passed through a 0.2 µm sterile syringe filter (Millipore) under aseptic conditions in a Class II laminar flow hood to remove any remaining particulates or aggregates. The filtrate was subsequently centrifuged under the same conditions to reconcentrate the beads, which were then resuspended in 50 µL of sterile Milli-Q water for subsequent functionalization.

Surface activation and enzyme conjugation were carried out using standard carbodiimide crosslinking chemistry. Briefly, 50 µL of the filtered particles suspension was added to 1 mL of freshly prepared activation buffer (50 mM MES, pH 5.5). To activate the surface carboxyl groups, 2 mM 1-ethyl-3-(3-dimethylaminopropyl)carbodiimide hydrochloride (EDC; Sigma-Aldrich) was added, and the suspension was incubated for 30 min at room temperature with gentle agitation. Subsequently, 100 µg of ATPase enzyme and 5 mM N-hydroxysuccinimide (NHS; Sigma-Aldrich) were added to the activated bead solution and incubated for 12 h at 4 °C to enable covalent coupling.

After conjugation, excess reagents and unbound enzyme were removed by ultrafiltration using a 300 kDa molecular weight cutoff (MWCO) centrifugal filter unit (Amicon Ultra, Millipore). The functionalized beads were washed twice with sterile PBS (pH 7.4) to ensure removal of residual reagents and finally resuspended in 50 µL of sterile PBS or phenol red-free medium for downstream applications and referred to as NP-A.

Enzyme loading and surface coverage estimation.

To estimate the enzyme loading and surface coverage of ATPase on 100 nm polystyrene nanoparticles, we combined geometric calculations with biochemical measurements. The surface

area of each spherical nanoparticle was calculated using $A = 4\pi r^2$, where $r = 50$ nm, yielding a surface area of approximately 31,400 nm² per particle.

The maximum number of ATPase molecules that could occupy the nanoparticle surface as a theoretical monolayer was estimated by assuming an average ATPase footprint of ~40 nm² per molecule (corresponding to an effective enzyme radius of ~3.5 nm, assuming a circular footprint, $A = \pi r^2$). Based on this estimate, a complete monolayer would contain approximately 785 ATPase molecules per nanoparticle.

The actual enzyme loading was determined experimentally by conjugating ATPase to carboxylate-modified nanoparticles using EDC/NHS chemistry and measuring the concentration of unbound ATPase in the supernatant by UV absorbance at 280 nm. Supernatant ATPase concentrations were determined by interpolation from a calibration curve of known ATPase standards measured under identical buffer conditions (**Figure S1**), confirming that all measurements fell within the validated linear detection range. The difference between the initial and unbound ATPase concentrations was used to calculate the amount of enzyme successfully conjugated to the particles (**Table S1, Figure S2**).

Using this depletion analysis, we estimated that each nanoparticle carried an average of ~1000 ATPase molecules, corresponding to an apparent surface coverage of ~130%. This slight oversaturation likely reflects uncertainties in the assumed enzyme footprint, partial multilayer formation, or non-upright (side-on) orientations of surface-bound ATPase molecules. The result simply suggests effective surface saturation.

The particle concentration of the final suspension was determined to be 3.6×10^{11} particles mL^{-1}, with a corresponding ATPase concentration of 70 µg mL^{-1}. These values indicate efficient enzyme conjugation and provide a basis for estimating the total number of catalytically active sites per unit volume of nanoparticle suspension.

4. Liposome preparation and ATPase functionalization

Liposomes were prepared from 75 mol% Egg PC, 20 mol% cholesterol, 4 mol% DSPE-PEG2000, and 1 mol% DSPE-PEG(2000)-NHS, with Rhodamine PE included for fluorescence labeling. Lipids were dried to a thin film, vacuum-desiccated, hydrated in sterile PBS (pH 7.4), and extruded through 100 nm polycarbonate membranes (21 passes) at 37 °C to form unilamellar vesicles. Freshly prepared NHS-functionalized liposomes were incubated with ATPase in PBS (pH 7.2–7.4) for 1–2 h at room temperature, allowing covalent coupling between NHS esters on the liposome surface and primary amines on ATPase. The reaction was quenched with Tris buffer, and unbound ATPase was removed by ultrafiltration. ATPase-decorated liposomes were stored at 4 °C and used within 24 h. Liposome size was confirmed by DLS measurements (**Figure S20**).

5. Fluorescence Correlation Spectroscopy (FCS)

For the fluorescence correlation spectroscopy (FCS) experiments, a 532 nm water-cooled pulsed laser (80 MHz, 100 ps) with an output power of 77 µW was used as the excitation source.[1] The laser beam was focused onto the sample using a 60x, 1.2 NA water-immersion objective, while the sample chamber was maintained at 37 °C with a temperature controller. Initial laser alignment and calibration were performed using a 2 µM Rhodamine B (RhB) solution. The fluorescence signal was recorded using a Time-Correlated Single Photon Counting (TCSPC) system (Becker & Hickl), and the resulting fluorescence intensity fluctuations were autocorrelated to analyze the dynamic properties of the fluorescent species.

Instrument calibration was performed using 50 nm carboxylate-modified fluorescent beads (Thermo Fisher Scientific) diluted at a 1:1000 ratio in phenol red-free medium, allowing for precise determination of the confocal volume and diffusion time constants. Experimental measurements were subsequently conducted using 100 nm orange-fluorescent nanoparticles (NP), pre-filtered as described in previous section, and diluted 1:1000 in PBS or phenol red-free culture medium.

Following this, cells were moved from the incubator and medium was changed to phenol red-free media (with or without particles). For each FCS experiment, the apical plasma membrane was first brought into focus using brightfield DIC microscopy at 60× magnification and defined as z = 0 µm. The focal plane was then shifted axially upward by the specified distance (+5 µm or +20 µm) using the microscope's calibrated z-drive, and FCS measurements were performed at that fixed axial position. Data were recorded over 150 minutes, with measurements acquired at 20-minute intervals, yielding eight time points per condition. The 5 µm and 20 µm measurements were conducted in separate experiments using independent, freshly prepared cell preparations. This was done for two reasons: (i) to ensure that data collection time points were synchronized across conditions - sequential measurement of both heights on the same cells would introduce a temporal offset between time points; and (ii) to avoid photobleaching and mechanical perturbation artifacts from repeated focal plane repositioning on the same cells. While the use of independent cell preparations introduces the possibility that batch-to-batch differences in confluency, cell health, or ATP release rate could contribute to apparent height-dependent differences, this concern is controlled by the NP-I and bare NP measurements, which were performed on the same independent preparations under identical conditions.

For lateral positioning, measurements in **Figure 2** (main text) were performed above the perinuclear cytoplasmic region, defined as the cytoplasmic area adjacent to but excluding the nucleus, identified in DIC images prior to each measurement session. This region was selected because it overlies the highest density of mitochondria in endothelial cells. Measurements in **Figure S7d-e** were performed above the cell periphery, defined as cytoplasmic regions near the cell edge while avoiding membrane protrusions. This region was chosen to test whether extracellular ATP released at the plasma membrane drives pericellular particle accumulation independently of mitochondrial proximity. In each case, the lateral position was fixed for the

duration of the experiment, and the axial height (+5 μm or +20 μm) was set relative to the local membrane position (z = 0 μm) defined at that lateral coordinate. A schematic overview of the FCS measurement geometry, including both lateral positions and axial heights, is provided in **Figure S7a.**

For independent repeat experiments, fresh cell preparations were used, and the focal plane was adjusted to +20 μm above the apical cell surface, while maintaining all other experimental parameters constant. Thus, the 5 μm and 20 μm measurements were conducted in separate experiments. Control measurements were performed under identical conditions in the absence of cells, using NP suspended in phenol red-free medium. Following the similar protocol, the FCS measurements were done with NP-A, and NP-I.

FCS analysis was performed to extract diffusion coefficients and local concentrations by fitting the autocorrelation curves with a one-component 3D diffusion model. Single-particle bursts were analyzed using BurstAnalyzer to quantify particle concentrations and estimate relative accumulation. All fits were evaluated for goodness-of-fit using residuals and chi-squared statistics.

6. Simulation of ATP release, gradient formation, and particle chemotaxis

We developed one-dimensional (1D) computational models using COMSOL to simulate ATP release from human aortic endothelial cells, and the subsequent ATP gradient formation and particle chemotaxis along the height direction (z direction) within the Bioptech cell culture dish (**Figure 2c** in main manuscript).

The 1D mass transfer equation of ATP can be written as:

$$\frac{\partial C_{ATP}}{\partial t} = D_{ATP}\frac{\partial^2 C_{ATP}}{\partial z^2} - \frac{k_{cat} C_P n_{EP} C_{ATP}}{K_M + C_{ATP}} \quad \text{(Eq. 1)}$$

Where C_{ATP} is the ATP concentration, D_{ATP} is the diffusion coefficient of ATP, k_{cat} is the catalytic turnover of ATPase, K_M is the Michaelis-Menten constant of ATPase, C_P is the ATPase-coated nanoparticle concentration, and n_{EP} is the mol of ATPase per particle.

The chemotaxis of ATPase-coated nanoparticle can be explained by phoretic flow mechanism, where a linear relationship exists between particle's chemotactic velocity and substrate gradient intensity ($U_{chemotaxis} \propto \nabla C_{substrate}$).[2–5] Consequently, the transport of ATPase-coated nanoparticles with chemotactic drift can be modeled using a cross-diffusion equation:

$$\frac{\partial C_P}{\partial t} = \frac{\partial}{\partial z}\left(D_P \frac{\partial C_P}{\partial z} - \alpha_{chemotaxis} C_P \frac{\partial C_{ATP}}{\partial z}\right) \quad \text{(Eq. 2)}$$

Where C_P is the particle number density, and D_P is the diffusion coefficient of the nanoparticle. Terms within the parentheses represent the total particle flux: the first term, $D_P \frac{\partial C_P}{\partial z}$, represents the diffusive flux based on Fick's law, while the second term, $\alpha_{chemotaxis} C_P \frac{\partial C_{ATP}}{\partial z}$, represents the drift

chemotaxis flux induced by the chemical gradient of ATP ($\frac{\partial C_{ATP}}{\partial z}$). The parameter $\alpha_{chemotaxis}$ is the chemotactic mobility coefficient. Gravity effect was assumed negligible, as no apparent particle settling was observed for bare nanoparticles case (**Figures S7b-c**).

To estimate the chemotactic mobility coefficient for the $100\ nm$ nanoparticles used in this study, a linear scaling analysis was performed based on the size-dependent reduction of viscous drag. In a previous study, the chemotactic mobility of $1\ \mu m$ sized urease-coated particles was determined to be $0.025\ \frac{\mu m/s}{\mu M/\mu m}$ using particle velocity and substrate gradient data.[2] As the particle diameter decreases from $1\ \mu m$ to $100\ nm$, the Stokes drag force decreases with the particle radius ($F_{drag} \propto R$), resulting in lower hydrodynamic resistance.[6] Assuming that the chemotactic driving force remains of similar magnitude, we estimated the mobility of $100\ nm$ particles by linearly scaling the mobility of $1\ \mu m$ particles:

$$\alpha_{this\ work} \approx \alpha_{previous\ work} \times \frac{R_{previous\ work}}{R_{this\ work}} = 0.25\frac{\mu m/s}{\mu M/\mu m} \quad \text{(Eq. 3)}$$

The above method provides only a rough approximation, as it neglects potential kinetic differences and additional size-dependent effects such as changes in enzyme number, surface interactions of different species, and enhanced rotational diffusion at the nanoscale.[3,7] Enzyme kinetics and surface loading are two important factors for the chemotactic response, as they influence the local reaction rate and the resulting chemical gradients, thereby affecting the particle's chemotactic mobility. As shown in **Figure 2a**, catalytically active ATPase-particles (NP-A, kcat ~ 48 s^{-1}) exhibit strong chemotactic accumulation near the cell surface, whereas inactive particles (NP-I and NP, kcat ~ 0 s^{-1}) remain evenly distributed over time. When comparing our system to previously reported urease-motor systems,[2] urease has a significantly higher catalytic turnover rate (kcat ~ 10^4 s^{-1}) compared to ATPase. Therefore, the current ATPase system would have a lower estimated mobility if considering kcat alone. However, different enzymatic systems also have different substrate-enzyme affinities (Km, where a lower Km indicates higher affinity). ATPases in our current system demonstrate a significantly higher substrate affinity (Km ~ 15 µM, **Table S2**) compared to urease (Km ~ 7.9 mM).[2] Because the effective reaction rate at experimental concentrations depends on both parameters, the exact quantitative relationship for kinetic scaling may require further investigation. Regarding enzyme surface loading, previous study found that self-propulsion velocity of enzyme-motors depends on enzyme loading in a non-linear fashion, with a threshold number of enzymes required for active motion, after which the speed tends to saturate.[8] Given the similar functionalization protocols used for the particles in the current ATPase system and previously reported urease systems, we anticipate that both systems have surface coverages well within this saturation regime and are sufficient to generate active motion. Minor variations in absolute surface loading above the threshold are unlikely to be the primary determining factor for differences in chemotactic mobility between the two systems. Overall, we used the hydrodynamic size-scaling as the primary characteristic estimate for chemotactic mobility calculation.

To calculate the diffusion distance and estimate the ATP release rate in the Bioptech cell culture dish, we referred to a human aortic endothelial cell as 60 μm long, 20 μm wide, and 5 μm thick.[9] The dish used for cell culture has a diameter of 23 mm and a solution volume of 1000 μL. The solution height (H, same as the ATP diffusion distance) can be calculated as:

$$H = \frac{V_{solution}}{\pi R^2} - h_{cell} \approx 2400\ \mu m \qquad \text{(Eq. 4)}$$

Table S3 summarizes all parameters used for simulation. For our experiments, system temperature is at 37 °C, estimated ATP release flux from cells ($J_{ATP,cell}$, assuming a constant release rate) is $2 \times 10^{-10}\ mol/(m^2 \cdot s)$, and the initial particle concentration (C_{P0}) is $3.6 \times 10^{11}\ [particles/mL]$. n_{EP} can be calculated using estimated number of enzyme molecules per particle ($N_{EP} = 1000\ [enzymes/particle]$) and Avogadro's number (N_A):

$$n_{EP} = \frac{N_{EP}}{N_A} = \frac{1000}{6.02 \times 10^{23}} = 1.7 \times 10^{-21}\ [mol/particle] \qquad \text{(Eq. 5)}$$

The 1D ATP and nanoparticle transport equation were solved together using COMSOL. For the ATP model, the initial ATP concentration was set to 0 for the entire domain. The $z = 0\ \mu m$ boundary, representing the surface where the cells are releasing ATP, was defined as a constant flux ($J_{ATP,cell}$) boundary condition. The $z = 2400\ \mu m$ boundary, which is the top of the cell culture dish solution, was defined as a non-flux boundary condition ($J_{ATP} = 0$). For nanoparticle chemotaxis model, the initial particle density (C_{P0}) was $3.6 \times 10^{11}\ [particles/mL]$ for the entire domain. The $z = 0\ \mu m$ and $z = 2400\ \mu m$ boundaries were no-flux boundaries, and particle loss due to cell uptake was neglected in the model. The 1D domain was discretized with a maximum mesh element size of 0.1 μm. Transient simulations were performed with an adaptive time step size.

Figure S8b shows simulated extracellular ATP concentration profile after 2 hours. We observed a steeper concentration gradient near the cell surface, with an effective distance of around 30 μm. As the gradient becomes weaker and finally approaches zero ($z > 30\ \mu m$), these nanoparticles cannot effectively perform chemotaxis due to strong Brownian motions. The rapid decay of the gradient occurs because cells release ATP at a relatively low rate under low-shear stress conditions, and ATPase-particles can effectively consume ATP. As a result, it creates a steeper gradient near cell surface over a shorter distance. The model (red line) captures the characteristic decay length observed in the experiments (blue line) (**Figure 2d** in manuscript). Specifically, a sharp accumulation was found near the cell surface, followed by relaxation to bulk particle number density levels beyond 30 μm.

7. Imaging and Analysis

Filtered particles (Ex/Em: 540/560 nm) were diluted 1:500 in phenol red-free medium and added to culture dishes containing adherent HAECs, followed by incubation under standard

conditions (37 °C, 5% CO_2). Imaging was performed at designated timepoints using a confocal fluorescence microscope (Leica TCS SP5) equipped with an HCX PL APO CS ×63.0/1.40 NA oil UV objective and 543 nm.

For organelle-specific imaging, live-cell staining was performed prior to fixation. Cells were incubated in phenol red-free medium containing organelle-specific dyes. Mitochondria were labeled using MitoTracker™ Deep Red FM (100 nM for 15 min; Ex/Em: 644/665 nm) and imaged using a 640 nm laser. Following staining, cells were washed with PBS and fixed in 4% paraformaldehyde for 15 min at room temperature. Fixed cells were washed twice with PBS and mounted using Fluoromount-G for imaging. All acquisition parameters, including laser power and exposure time, were kept constant across experimental conditions. The imaging was performed at Leica microscope with 60x oil objective and 530 nm laser.

Colocalization analysis was performed using FIJI (ImageJ). Fluorescence channels were independently thresholded to minimize background, and ROIs were manually defined over individual cells. Pearson's correlation coefficient (PCC) was calculated using the Coloc2 plugin to quantify colocalization between particles and organelles. A minimum of 10 cells per condition was analyzed, and PCC values were reported as mean ± s.d.

To assess whether internalized NP-A particles are retained through the endo-lysosomal pathway, HAECs were incubated with NP-A for 2 hours under standard conditions (37 °C, 5% CO_2). Following incubation, cells were stained with LysoTracker Red DND-99 (75 nM, 30 min; Ex/Em: 577/590 nm) in phenol red-free medium at 37 °C to label lysosomes. Cells were then washed twice with PBS and imaged using a fluorescence microscope equipped with a 60× objective under identical acquisition settings. For higher-resolution imaging, a reduced NP-A concentration was used to resolve individual particle puncta. Colocalization between NP-A (cyan) and LysoTracker (red) was quantified using the Coloc2 plugin in FIJI (ImageJ) (**Figure S10**). Pearson's correlation coefficient (PCC) was calculated from a minimum of 10 cells per condition.

Imaging with liposomes. Rhodamine PE labeled ATPase-liposomes (Ex/Em: 540/560 nm) were diluted 1:5 in phenol red-free medium and added to HAEC cultures, followed by incubation under standard conditions. After 2 hours, cells were incubated with MitoTracker™ Deep Red FM (100 nM, 15 min; Ex/Em: 644/665 nm) in phenol red-free medium at 37 °C and 5% CO_2 for organelle-specific labeling. Following staining, cells were washed with PBS and imaged using a fluorescence microscope equipped with a 60× water-immersion objective and chroma filters (Ex: 535/50 nm, Em: 610/75 nm). Imaging was performed at designated timepoints under identical acquisition settings.

8. Flow Cytometry

For flow cytometry analysis, endothelial cells were seeded in culture dishes and grown to ~70 - 80% confluency under standard conditions. Fluorescent particles were washed with PBS to remove

the storage buffer and resuspended in cell culture medium at the appropriate concentration. Cells were incubated with particle-containing medium for 1 hour to allow bead uptake, followed by gentle washing with PBS (2-3 times) to remove unbound beads.

Cells were dissociated using Accutase and neutralized with serum-containing medium. Cell suspensions were collected by centrifugation at 300 × g for 3 min at 4 °C, washed twice with PBS. Prior to acquisition, cells were resuspended in flow cytometry buffer.

Flow cytometry was performed on a BD LSR (Becton Dickinson Laser Scanning Research) Fortessa cytometer. Particles were detected using a 532 nm (150 mW) green laser with a 575/25 nm bandpass filter. Single-stained controls for particles were used for compensation. Gating was performed using forward and side scatter (FSC/SSC) to exclude debris and cell doublets. Fluorescence-positive populations were identified using single-parameter histograms and two-dimensional dot plots. Events were classified based on bead-positive cells. Data were acquired for a minimum of 10,000 events per sample and analyzed using FlowJo software (BD, Becton Dickinson).

9. Inhibitor Treatments and Starvation Protocol

For metabolic inhibition studies, HAECs were incubated with either Oligomycin A (OA, 2-10 µM) or Sodium orthovanadate (NV, 2-10 µM). For Oligomycin A treatment, cells were preincubated with the inhibitor for 30 min at 37 °C in phenol red-free medium to inhibit mitochondrial ATP synthase activity. For orthovanadate treatment, cells were incubated for 30 min under the same conditions to inhibit plasma membrane ATPases. In both cases, inhibitor-containing medium was maintained throughout subsequent bead incubation or staining procedures to preserve inhibition effects. Control treatments were performed in parallel using vehicle controls: DMSO (final concentration matched to that of Oligomycin A treatment) and PBS for orthovanadate-treated samples.

For starvation experiments, cells were washed twice with PBS and incubated in glucose-free and serum-free DMEM for 4 h at 37 °C in a humidified 5% CO_2 incubator. Where indicated, starvation was combined with bead incubation or organelle staining during the final hour of the starvation period. Control cells were maintained in complete medium under standard conditions.

10. ATP Release Measurement and Protein Normalization

To quantify extracellular ATP release, HAECs were cultured in 24-well plates and subjected to specific treatments, including standard conditions, starvation (glucose- and serum-free medium), or metabolic inhibition using Oligomycin A (2-10 µM) or Na_3VO_4 (2-10 µM). After the treatment period, 1000 µL of phenol red-free medium was added to each well and incubated under standard conditions. At the end of incubation, the medium was carefully collected into fresh tubes, and ATP

concentration was determined using a luciferin-luciferase bioluminescence assay (ATP Determination Kit, Molecular Probes, A22066), according to the manufacturer's instructions.

The reaction was based on firefly luciferase-catalyzed ATP-dependent oxidation of D-luciferin, generating luminescence with peak emission at ~560 nm. The working reaction solution was prepared by combining 20× reaction buffer, D-luciferin, DTT, and recombinant luciferase in deionized water. ATP standards (1 nM to 1 μM) were prepared by serial dilution from a 5 mM ATP stock. Sample volumes were adjusted to remain below 10% of the total 100 μL reaction volume. Luminescence was recorded immediately after sample addition using a microplate luminometer, and ATP concentrations were interpolated from the standard curve (**Figure S5**). Background luminescence was subtracted, and all samples were measured in triplicates.

After media collection, cells in each well were washed twice with PBS to eliminate residual extracellular ATP. For protein normalization, cells were lysed directly in the same wells using 100 μL of RIPA buffer (50 mM Tris-HCl, pH 7.5; 150 mM NaCl; 1% Triton X-100; 0.1% SDS; protease inhibitors). Lysates were incubated on ice for 20–30 min with occasional vortexing for complete lysis. Samples were centrifuged at 12,000 × g for 10 min at 4 °C, and the supernatant was collected for protein quantification.

Protein concentration was measured using a BCA assay (Bicinchoninic Acid Assay, Thermo Scientific). The BCA working reagent (WR) was freshly prepared by mixing Reagents A and B in a 50:1 ratio. BSA standards (0-500 μg/mL) were used to generate the standard curve. 25 μL of each sample or standard was added to a 96-well plate, followed by 200 μL of WR, and incubated at 37 °C for 30 min. Absorbance was measured at 562 nm, and protein concentrations were calculated from the standard curve (**Figure S12**). All measurements were performed in triplicate.

The same ATP release measurement and protein normalization protocol was applied to HeLa cells cultured under nutrient-rich conditions for cross-cell-type comparison of extracellular ATP levels.

ATP Normalization. ATP concentrations (in nanomolar) obtained from the standard curve were normalized to total protein concentration in each corresponding lysate sample. Final values were expressed as:

$$Normalized\ ATP = \frac{ATP\ concentration\ (nM)}{Total\ protein\ (mg)}$$

All values were reported as nM ATP per mg protein (or as pmol/μg), allowing for comparison across treatments independent of variations in cell number or protein content.

11. MTT assay

Cells were seeded into 96-well tissue culture plates at a density of 5,000-10,000 cells per well and incubated under standard conditions (37 °C, 5% CO_2) for 48-72 h to reach optimal confluency. After experimental treatments, 10 μL of 12 mM MTT stock solution was added to each well

containing 100 µL of culture medium. Plates were gently mixed and incubated at 37 °C for 4 h to allow intracellular reduction of MTT to formazan.

After incubation, the medium was removed, leaving 25 µL in each well. DMSO (Dimethyl sulfoxide, 50 µL) was added directly to each well to dissolve the formazan crystals, and the mixture was thoroughly pipetted to ensure homogeneity. Plates were incubated at 37 °C for 10 min before absorbance measurement. Absorbance was recorded at 540 nm using a plate reader. Blank wells containing medium and MTT without cells were used for background correction.

12. Data Analysis and Normalization.

Cell viability was calculated by normalizing absorbance values against untreated control wells, using the following formula:

$$Percent\ Viability = \left(\frac{Abs_{sample} - Abs_{blank}}{Abs_{control} - Abs_{blank}}\right) x\ 100$$

where Abs_{sample} is the absorbance of treated cells, $Abs_{control}$ is the absorbance of untreated cells, and Abs_{blank} is the background absorbance from wells without cells.

All measurements were performed in triplicates, and data are presented as mean ± s.d. across replicates.

13. Statistical analysis.

All data are presented as mean ± standard deviation unless otherwise stated. Statistical comparisons between NP-A and NP or NP-I were performed using two-sided Student's t-tests, unless otherwise indicated in the figure captions. For Figures 2a and 2b, statistical significance was determined using two-way ANOVA with time and particle type as factors, followed by Šídák's multiple-comparisons test comparing NP-A with NP and NP-I at each time point. Statistical significance was assigned as ns, $p \geq 0.05$; *$p < 0.05$; **$p < 0.01$; and ***$p < 0.001$. Statistical analyses were performed using GraphPad Prism.

C. Supplementary Figures S1-S20

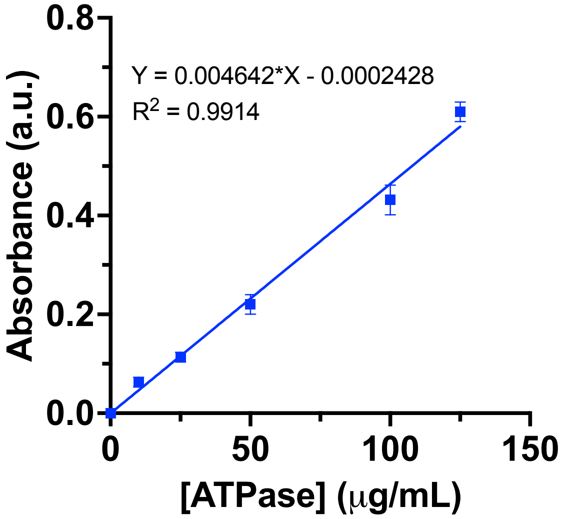

Figure S1. Calibration curve obtained by measuring absorbance at 280 nm as a function of known ATPase concentrations under identical assay conditions. Data represent mean ± SD (n = 3). The linear fit was used to determine ATPase concentration in subsequent experiments.

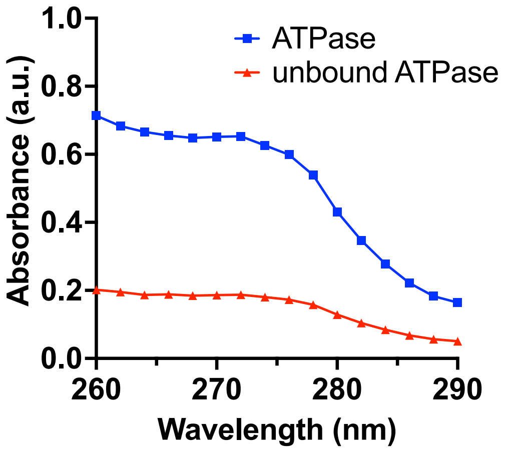

Figure S2. UV absorbance measurements showing the initial ATPase concentration (total ATPase added prior to conjugation) and the concentration of unbound ATPase remaining in the supernatant after EDC/NHS conjugation to nanoparticles. The difference between the initial and unbound ATPase concentrations was used to calculate the amount of ATPase conjugated per particle and estimate surface coverage. Data shown were used for enzyme loading calculations reported in Table S1.

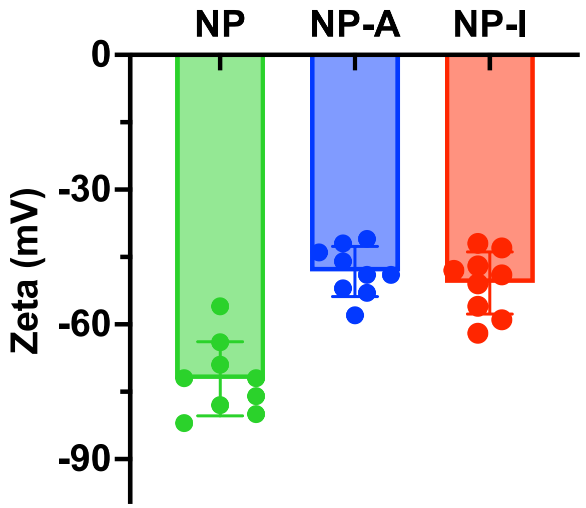

Figure S3. Zeta potential plots for the carboxylate functionalized particles (NP, green), ATPase-coated particles (NP-A, blue), and inactive ATPase-coated particles (NP-I, red) suggesting efficient coating of ATPase over nanoparticle surface and the surface potential does not change on heating the particles (for NP-I). Experimental condition: [NP] = [NP-A] = [NP-I] = 1 μL/mL in PBS at 25 ºC.

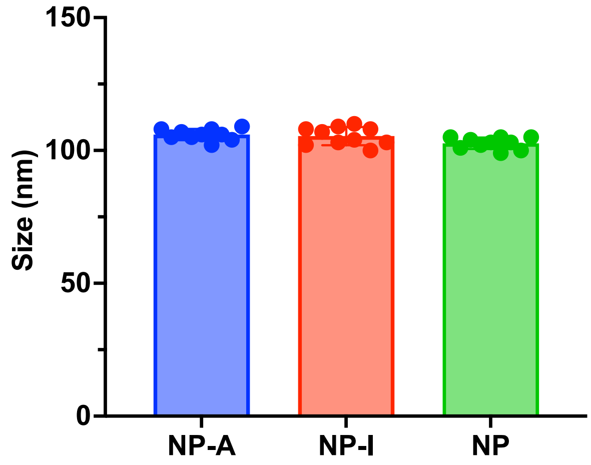

Figure S4. Hydrodynamic diameter for the carboxylate functionalized particles (NP, green), ATPase-coated particles (NP-A, blue), and inactive ATPase-coated particles (NP-I, red). Experimental condition: [NP] = [NP-A] = [NP-I] = 1 μL/mL in PBS at 25 ºC.

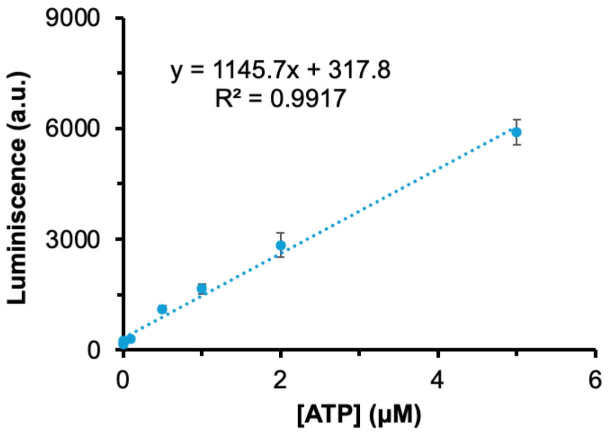

Figure S5. Standard curve for ATP quantification using luciferase assay. Luminescence shows a strong linear correlation with ATP concentration ($R^2 = 0.9917$), enabling accurate quantification in experimental samples. Data represent mean ± SD from n = 3 independent experiments.

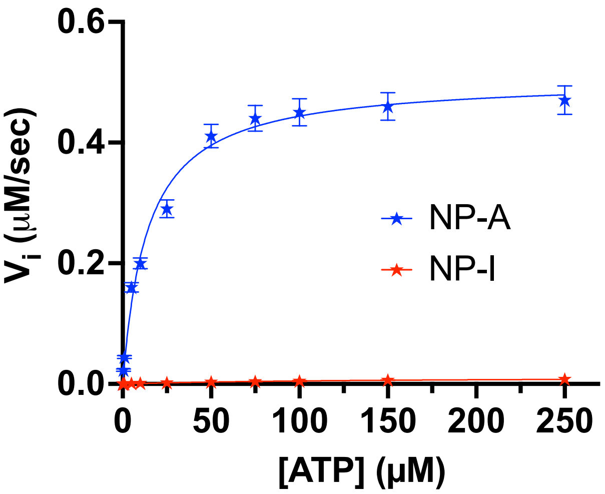

Figure S6. Michaelis-Menten plot for the ATPase activity in media at 25 °C using luciferase assay. The enzymatic activity was measured with active ATPase-coated particles (NP-A) and inactive ATPase-coated particles (NP-I) while maintaining the concentration of ATPase on particle surface at 10 nM. The curve demonstrates classical Michaelis-Menten behavior, indicating substrate-dependent catalytic turnover. Data sets are presented as mean ± standard deviation from triplicate measurements.

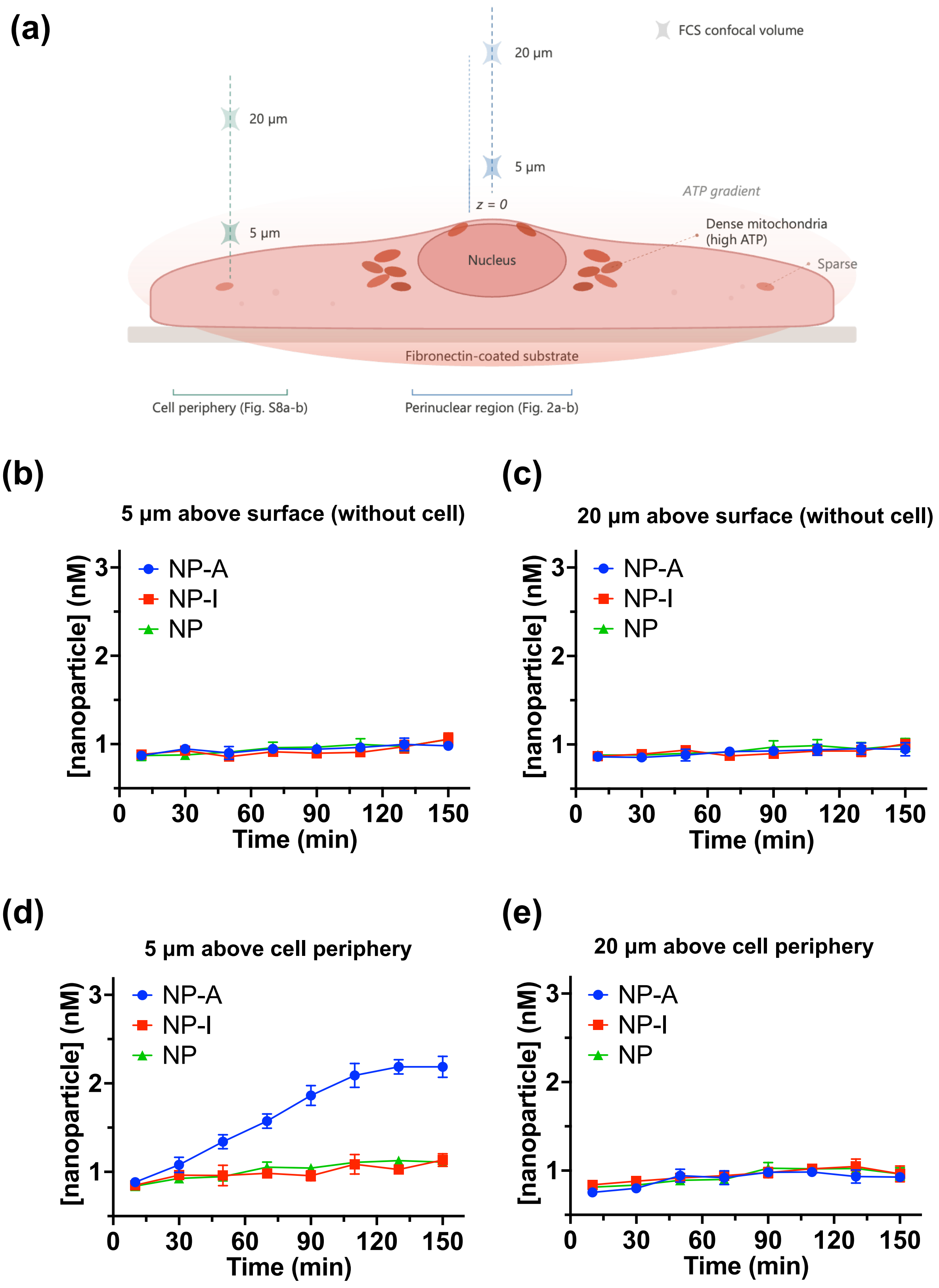
(a)
FCS confocal volume
20 μm
5 μm
20 μm
5 μm
z = 0
ATP gradient
Dense mitochondria (high ATP)
Sparse
Nucleus
Fibronectin-coated substrate
Cell periphery (Fig. S8a-b)
Perinuclear region (Fig. 2a-b)
(b)
5 μm above surface (without cell)
(c)
20 μm above surface (without cell)
(d)
5 μm above cell periphery
(e)
20 μm above cell periphery
NP-A
NP-I
NP
[nanoparticle] (nM)
Time (min)

Figure S7. (a) Schematic of FCS measurement geometry. Side-view of an adherent HAEC showing the two lateral positions used for FCS: the perinuclear region (blue; Figure 2a-b), selected for its high mitochondrial density, and the cell periphery (green; Figure S7b-c). At each position, the apical membrane was defined as z = 0 µm, and measurements were acquired at +5 µm and +20 µm. Hourglass shapes indicate the confocal detection volume. Red ellipses represent mitochondria; the surrounding gradient depicts the extracellular ATP concentration field. FCS analysis of NP-A, NP-I, and NP concentrations over time. (b, c) Under cell-free conditions, NP-A shows no differential accumulation at either height, confirming that cellular ATP release is required for localized enrichment. (d) At 5 µm above the cell surface in media, NP-A shows a pronounced time-dependent increase in local concentration, unlike NP-I and NP controls. (e) At 20 µm, all groups remain largely unchanged, indicating that gradient-driven accumulation is restricted to the pericellular region. Data represent mean ± SD from n = 3 independent experiments.

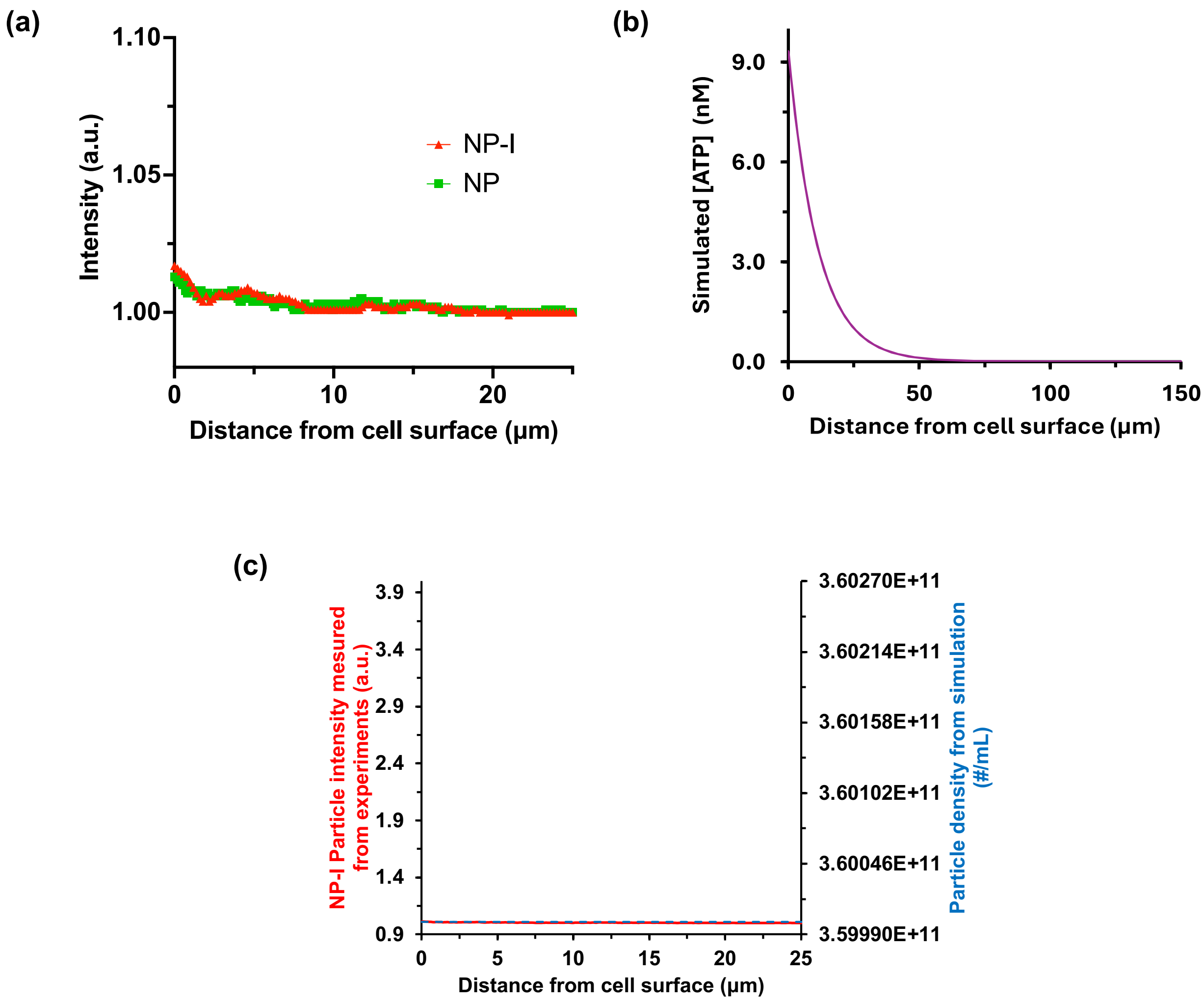

Figure S8. (a) Z-stack intensity profile of NP-I, and NP obtained from structured illumination microscopy (SIM). Fluorescence intensity across axial slices was quantified using ImageJ, revealing NP-I and NP show uniform and low-intensity distributions, indicating limited axial localization. (b) Simulated extracellular ATP concentration profile after 2 hours, showing a steep gradient localized near the cell surface that decays with distance into the bulk medium. (c) Representative particle fluorescence intensity profile (red solid trace) extracted from SIM z-stack imaging for NP-I, showing no enhanced signal near the cell surface. The blue dotted curve shows the corresponding simulated particle density profile as a function of distance from the cell surface and matches the experimental profile.

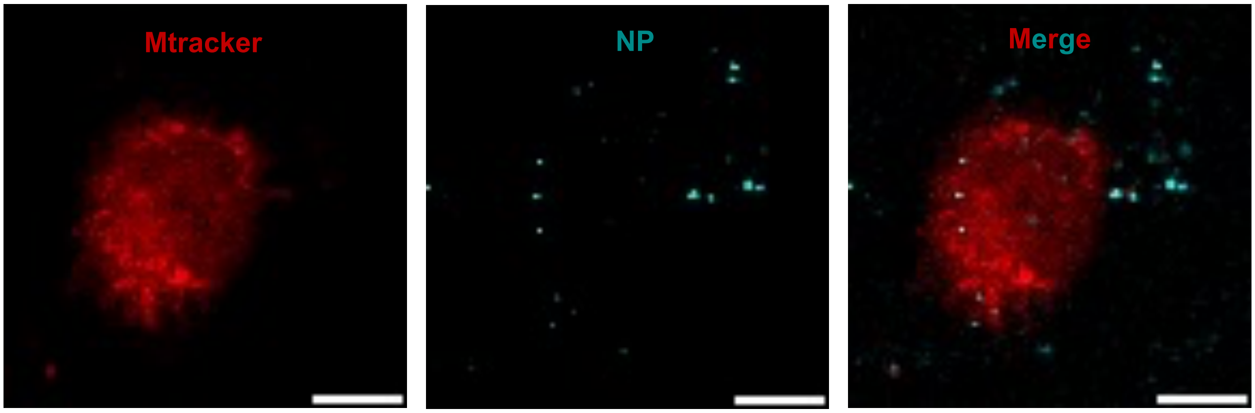

Figure S9. Confocal microscopy images showing minimal colocalization of bare nanoparticles (NP, cyan) with mitochondria labeled by MitoTracker Deep Red (red). Scale bars = 10 µm.

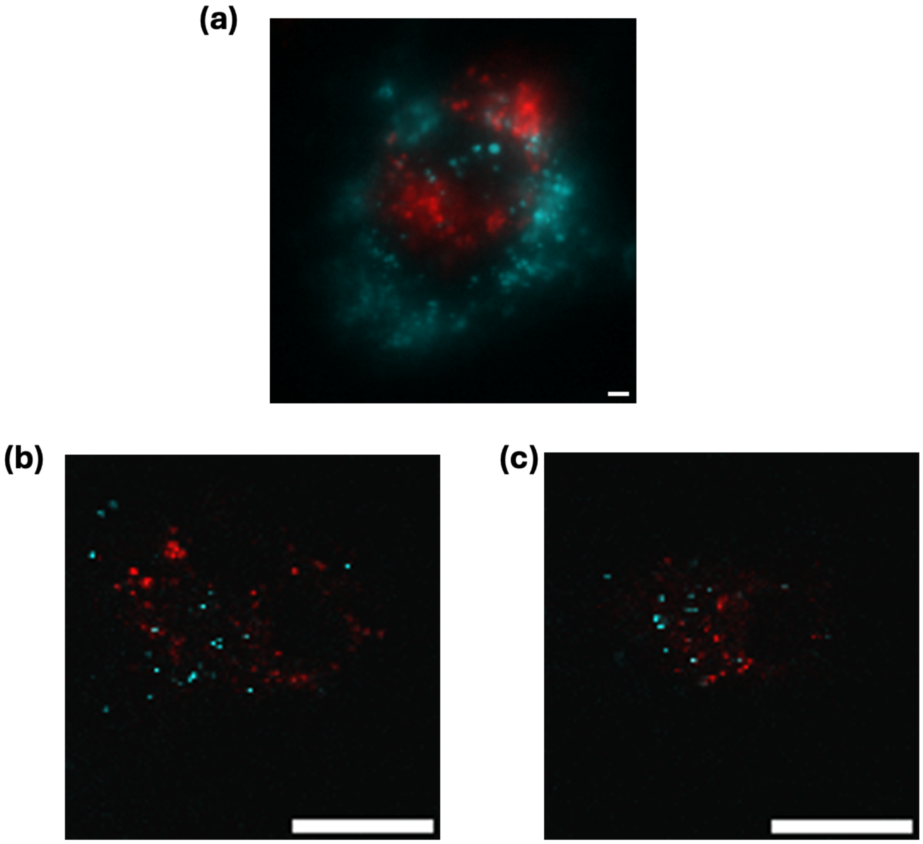

Figure S10. NP-A does not colocalize with lysosomes following cellular internalization. (a) Widefield fluorescence image of HAECs showing merged NP-A (cyan) and LysoTracker (red) signals. The cyan signal forms a peripheral distribution surrounding lysosomal puncta, indicating spatial segregation. (b, c) Confocal fluorescence images acquired at reduced NP-A concentration to resolve individual particle puncta. Cyan (NP-A) and red (LysoTracker) signals are largely non-overlapping, yielding a Pearson's correlation coefficient of r = 0.07, confirming minimal lysosomal association of internalized NP-A. Scale bars = 20 μm.

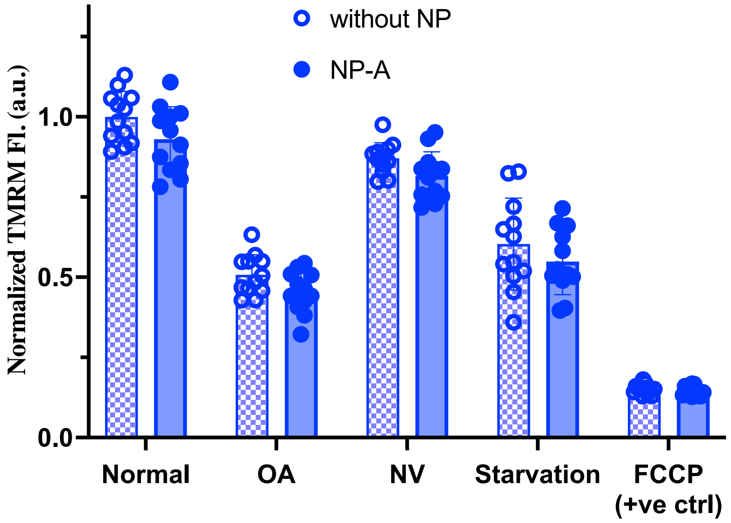

Figure S11. Quantification of mitochondrial membrane potential using TMRM (Tetramethylrhodamine methyl ester) under various treatment conditions. Cells were stained with TMRM to assess mitochondrial membrane potential following different treatments. Bar graphs represent mean TMRM fluorescence intensity normalized to normal cells. Solid bars indicate NP-A-treated groups, while patterned bars represent cells without particles. Data represent mean ± standard deviation from 12 independent experiments per condition.

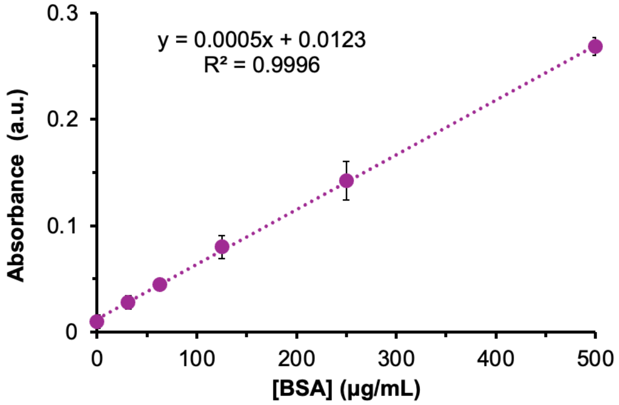

Figure S12. Standard curve for protein quantification using BSA. Absorbance shows a strong linear correlation with BSA concentration ($R^2 = 0.9996$), enabling accurate protein quantification in experimental samples.

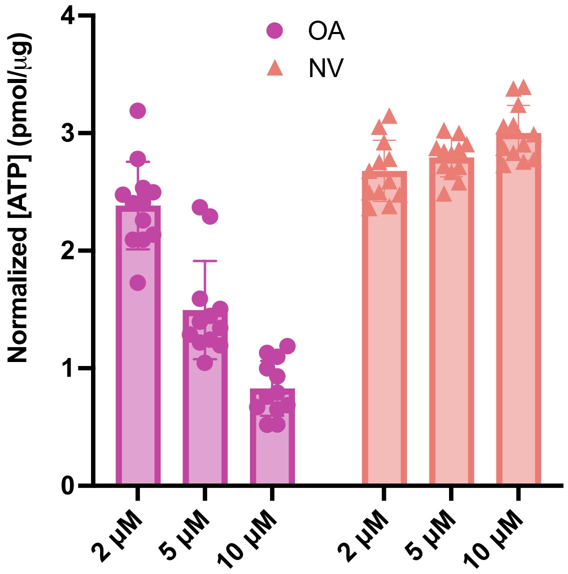

Figure S13. Quantification of extracellular ATP levels under oligomycin and sodium orthovanadate treatment using luciferase-based assay. ATP concentrations were measured in cells treated with varying concentrations of oligomycin (left, magenta) and sodium orthovanadate (right, orange). Oligomycin treatment led to a dose-dependent reduction in ATP levels, consistent with mitochondrial ATP synthase inhibition. In contrast, sodium orthovanadate treatment did not significantly alter ATP levels across tested concentrations. Bars represent mean ATP levels with individual replicates shown as circles (oligomycin) and triangles (orthovanadate). Data represent mean ± standard deviation from 12 independent experiments.

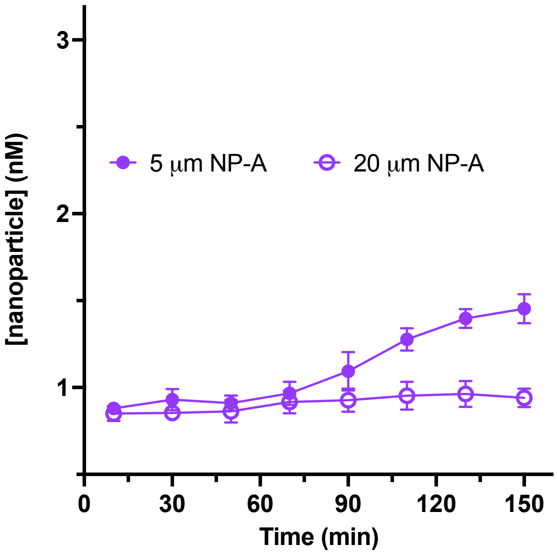

Figure S14. Time-dependent nanoparticle concentration profiles measured using FCS at different heights for NP-A (ATPase coated nanoparticles) under starving conditions. Measurements were taken at 5 μm and 20 μm heights from the cell surface. Data are presented as mean ± standard deviation from three independent measurements.

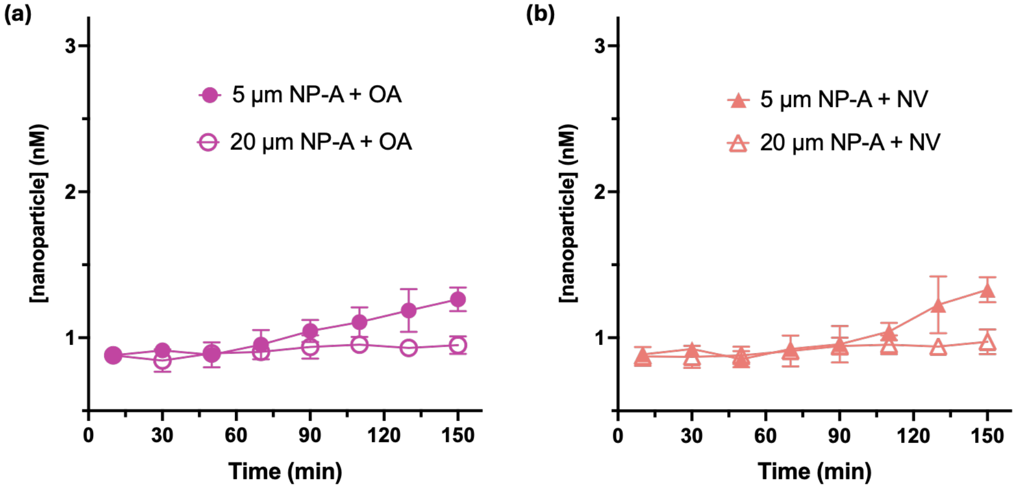

Figure S15. Time-resolved nanoparticle localization under metabolic inhibition. Fluorescence correlation spectroscopy (FCS) measurements of NP-A (ATPase-coated nanoparticles) concentrations at 5 µm and 20 µm above the cell surface following treatment with 10 µM oligomycin (OA, left) and 10 µM sodium orthovanadate (NV, right). Under both conditions, modest accumulation of NP-A was observed at 5 µm, while concentrations at 20 µm remained largely unchanged, suggesting diminished or abolished gradient-driven localization. Data represent mean ± SD from three independent experiments.

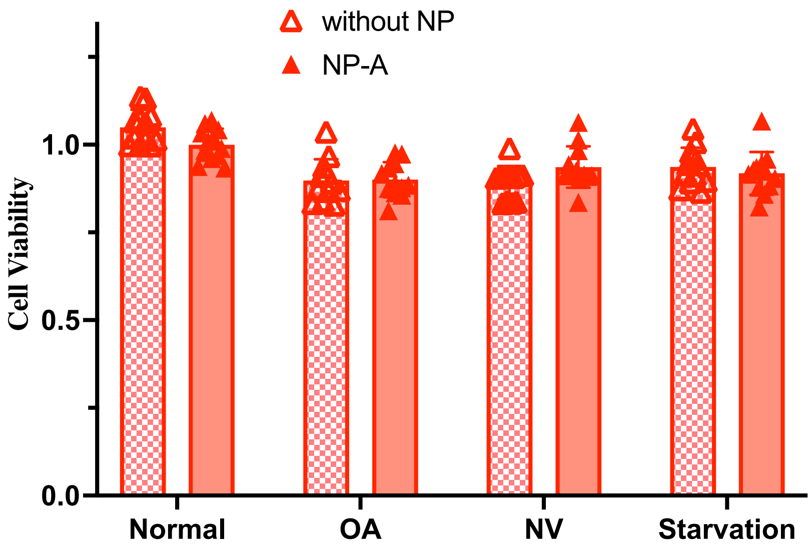

Figure S16. MTT (3-(4,5-dimethylthiazol-2-yl)-2,5-diphenyltetrazolium bromide) assay quantifying cell viability under various treatment conditions with and without NP-A. Comparable viability across most conditions indicates that NP-A does not induce significant cytotoxicity under the tested conditions. Data represent mean ± standard deviation from 12 independent experiments.

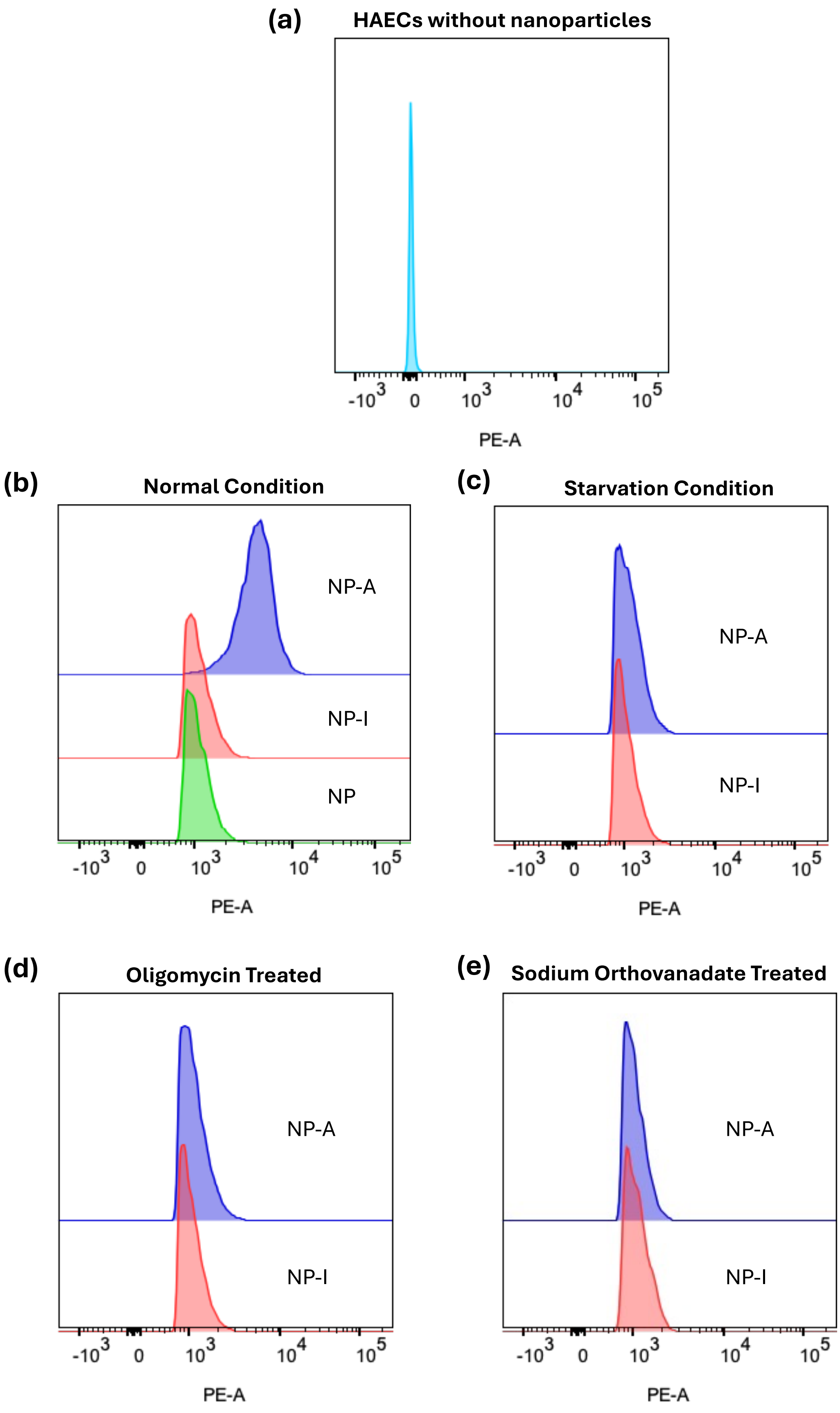
(a)
HAECs without nanoparticles
-10^3
0
10^3
10^4
10^5
PE-A
(b)
Normal Condition
NP-A
NP-I
NP
-10^3
0
10^3
10^4
10^5
PE-A
(c)
Starvation Condition
NP-A
NP-I
-10^3
0
10^3
10^4
10^5
PE-A
(d)
Oligomycin Treated
NP-A
NP-I
-10^3
0
10^3
10^4
10^5
PE-A
(e)
Sodium Orthovanadate Treated
NP-A
NP-I
-10^3
0
10^3
10^4
10^5
PE-A

Figure S17. Representative flow cytometry histograms showing nanoparticle uptake by HAECs under different metabolic conditions. (a) Unstained cells (no particles) showing baseline autofluorescence. (b) Cells incubated under nutrient-rich conditions with NP-A (blue), NP-I (red), and bare NP (green). NP-A exhibits a sharp, right-shifted fluorescence peak indicating uniform, high particle uptake, whereas NP-I and NP show lower fluorescence. (c) Glucose starvation, (d) oligomycin A (10 μM), and (e) sodium orthovanadate (10 μM) conditions showing NP-A (blue) and NP-I (red). Under all perturbation conditions, NP-A distributions broaden and shift leftward toward NP-I, while NP-I remains in a consistent position across all conditions, confirming that endocytic capacity is preserved. Histograms are normalized to mode. PE-A fluorescence corresponds to nanoparticle signal (Ex/Em: 540/560 nm).

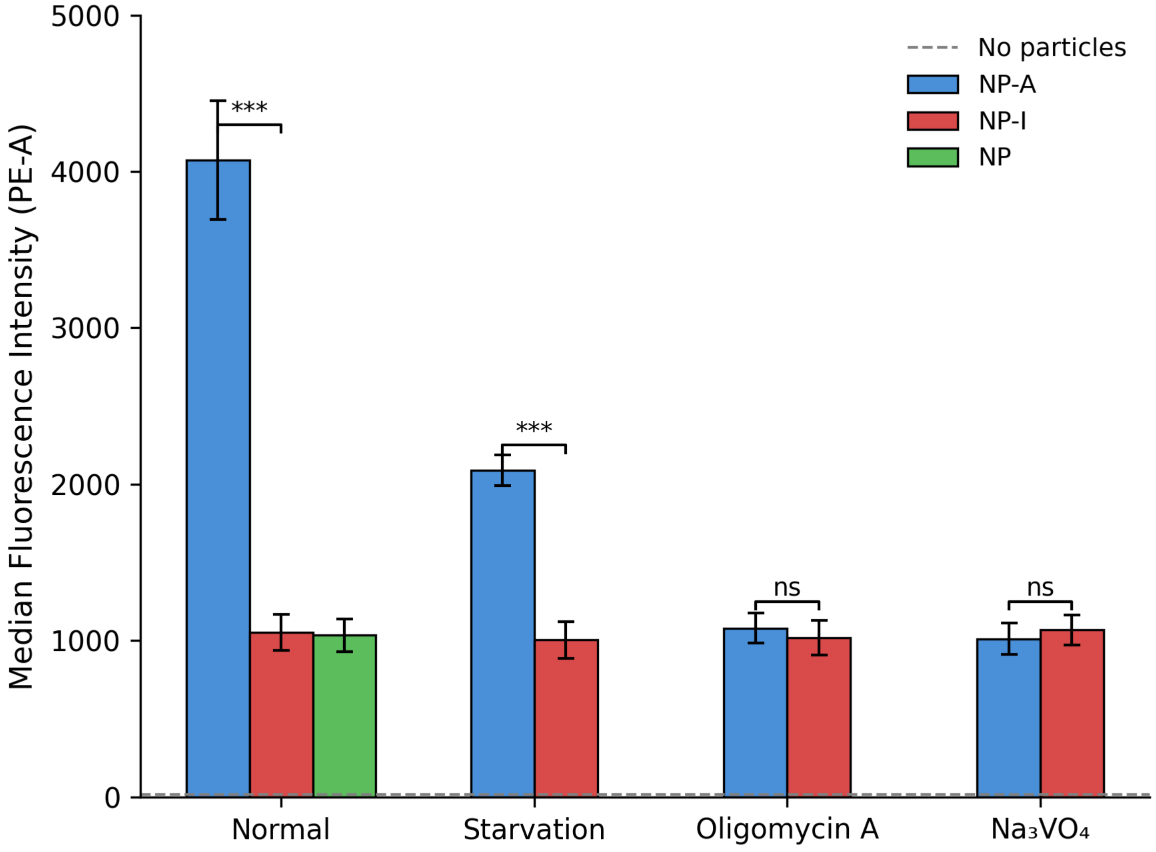

Figure S18. Quantification of nanoparticle uptake by flow cytometry under different metabolic conditions. Median fluorescence intensity (MFI, PE-A) for NP-A (blue), NP-I (red), and bare NP (green, normal condition only). Under nutrient-rich conditions, NP-A exhibits significantly higher uptake compared to NP-I and NP. Glucose starvation moderately reduces NP-A uptake, while oligomycin A and sodium orthovanadate treatment reduce NP-A uptake to levels statistically indistinguishable from NP-I. NP-I MFI remains unchanged across all conditions, confirming that metabolic perturbations do not impair cellular endocytic capacity. Dashed line indicates baseline fluorescence of cells without particles. Data represent mean ± SD from n = 3 independent experiments. Statistical significance between NP-A and NP-I was assessed using a two-sided Student's t-test (***$p < 0.001$; ns, not significant, $p \geq 0.05$).

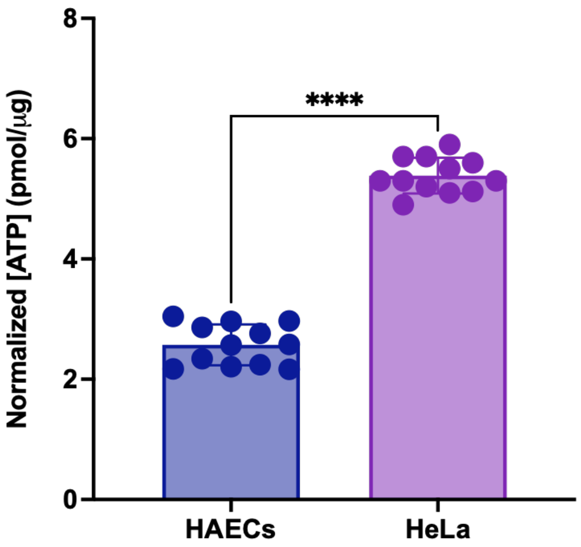

Figure S19. Comparison of extracellular ATP release between HAECs and HeLa cells under nutrient-rich conditions. ATP concentrations were measured using a luciferase-based bioluminescence assay and normalized to total cellular protein determined by BCA assay. HeLa cells released approximately 2-fold higher extracellular ATP compared to HAECs, consistent with the elevated metabolic activity of cancer cells. Bars represent mean ± standard deviation. Statistical significance was assessed using a two-sided Student's t-test ($p < 0.0001$ (****)). Data represent mean ± SD from $n = 12$ independent experiments.

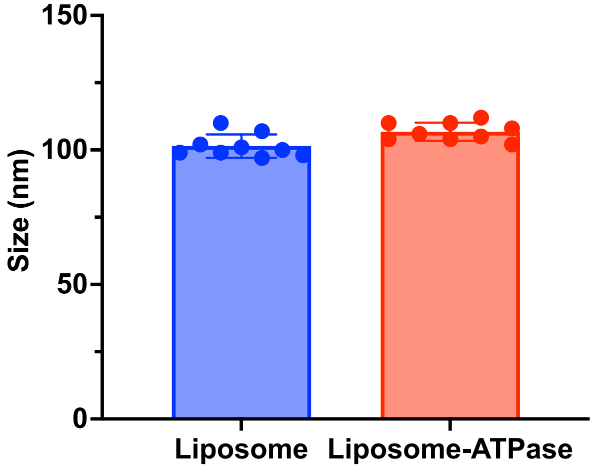

Figure S20. Hydrodynamic diameter for the liposome (blue), ATPase-coated liposomes (red).

D. Supplementary Table S1-S4

Table S1. Physicochemical parameters for ATPase-coated nanoparticles

Parameter	Value
Particle diameter (nm)	100
Surface area per particle (nm^2)	31,400
Estimated ATPase footprint (nm^2)	40
Monolayer ATPase capacity (molecules/particle)	785
Loaded ATPase (molecules/particle)	1000
Coating percentage (%)	130%
Particle concentration (particles/mL)	3.6×10^{11}
Bound ATPase concentration (μg/mL)	70

Table S2. Kinetic parameters for nanoparticle-bound ATPase using Luciferase assay suggesting NP-A retains enzymatic activity post-conjugation.

Parameter	NP-A
V_{max} (nM/sec)	480
K_m (μM)	15
Total enzyme concentration [E] (nM)	10
k_{cat} (sec^{-1})	48

Table S3. Parameters used for simulation

Parameter	Description	Value	Source
D_{ATP}	Diffusion coefficient of ATP	2.4×10^{-10} $[m^2/s]$	[10]
D_P	Diffusion coefficient of nanoparticles	4.4×10^{-12} $[m^2/s]$	Stokes-Einstein equation: $\frac{k_B T}{6\pi\eta r}$
$J_{ATP,cell}$	ATP release flux from cells	2×10^{-10} $[mol/(m^2 \cdot s)]$	From experiments
N_{EP}	Number of enzyme molecules per particle	1000 $[enzymes/particle]$	From experiments
n_{EP}	Mol of enzymes per particle	1.7×10^{-21} $[mol/particle]$	$\frac{N_{EP}}{N_A}$
C_{P0}	Initial particle number density	3.6×10^{11} $[particles/mL]$	From experiments
k_{cat}	Catalytic turnover of ATPase	48 $[s^{-1}]$	From experiments
K_M	Michaelis-Menten constant	15 $[\mu M]$	From experiments
H	Total diffusion distance (Petri dish solution height)	2400 $[\mu m]$	From experiments
$\alpha_{chemotaxis}$	Chemotactic mobility coefficient of nanoparticles	$0.25 \frac{\mu m/s}{\mu M/\mu m}$	Based on the scaling analysis

Table S4. Pearson's correlation analysis of mitochondrial colocalization for NP-A and NP-I under various metabolic conditions and across cell types. NP-A exhibits the highest colocalization in nutrient-rich HAECs and HeLa cells, with HeLa showing significantly higher colocalization than HAECs ($p < 0.01$), which decreases under starvation and metabolic inhibition in HAECs. NP-I shows consistently low and statistically indistinguishable colocalization across both cell types ($p = 0.39$, not significant), indicating that targeting is dependent on enzymatic activity rather than cell morphology. Statistical comparisons between cell types were performed using two-sided Student's t-tests.

Condition	Pearson's Correlation Coefficient (r)
HAECs + NP-A	0.55 ± 0.07
HAECs + NP-I	0.19 ± 0.02
Starving HAECs + NP-A	0.35 ± 0.03
OA Treated HAECs + NP-A	0.15 ± 0.02
NV Treated HAECs + NP-A	0.18 ± 0.02
HeLa cells + NP-A	0.63 ± 0.05
HeLa cells + NP-I	0.2 ± 0.03

E. References

(1) Gullapalli, R. R.; Tabouillot, T.; Mathura, R.; Dangaria, J. H.; Butler, P. J. Integrated Multimodal Microscopy, Time-Resolved Fluorescence, and Optical-Trap Rheometry: Toward Single Molecule Mechanobiology. *J. Biomed. Opt.* **2007**, *12* (1), 014012. https://doi.org/10.1117/1.2673245.
(2) Sapre, A.; Lu, X.; Tseng, Y.-C.; Mansour, M.; Mandal, N. S.; Sen, A. Non-Reciprocal Chemotactic Movement in Enzyme Cascade under Flow-Free Conditions. *Cell Rep. Phys. Sci.* **2025**, *6* (7), 102666. https://doi.org/10.1016/j.xcrp.2025.102666.
(3) Popescu, M. N.; Uspal, W. E.; Bechinger, C.; Fischer, P. Chemotaxis of Active Janus Nanoparticles. *Nano Lett.* **2018**, *18* (9), 5345–5349. https://doi.org/10.1021/acs.nanolett.8b02572.
(4) Ouazan-Reboul, V.; Agudo-Canalejo, J.; Golestanian, R. Self-Organization of Primitive Metabolic Cycles Due to Non-Reciprocal Interactions. *Nat. Commun.* **2023**, *14* (1), 4496. https://doi.org/10.1038/s41467-023-40241-w.
(5) Shandilya, E.; Rallabandi, B.; Maiti, S. In Situ Enzymatic Control of Colloidal Phoresis and Catalysis through Hydrolysis of ATP. *Nat. Commun.* **2024**, *15* (1), 3603. https://doi.org/10.1038/s41467-024-47912-2.
(6) Jia, H.; Zhu, G.; Wang, P. Catalytic Behaviors of Enzymes Attached to Nanoparticles: The Effect of Particle Mobility. *Biotechnol. Bioeng.* **2003**, *84* (4), 406–414. https://doi.org/10.1002/bit.10781.
(7) Arqué, X.; Romero-Rivera, A.; Feixas, F.; Patiño, T.; Osuna, S.; Sánchez, S. Intrinsic Enzymatic Properties Modulate the Self-Propulsion of Micromotors. *Nat. Commun.* **2019**, *10* (1), 2826. https://doi.org/10.1038/s41467-019-10726-8.
(8) Patiño, T.; Feiner-Gracia, N.; Arqué, X.; Miguel-López, A.; Jannasch, A.; Stumpp, T.; Schäffer, E.; Albertazzi, L.; Sánchez, S. Influence of Enzyme Quantity and Distribution on the Self-Propulsion of Non-Janus Urease-Powered Micromotors. *J. Am. Chem. Soc.* **2018**, *140* (25), 7896–7903. https://doi.org/10.1021/jacs.8b03460.
(9) Félétou, M. *The Endothelium, Part I: Multiple Functions of the Endothelial Cells -- Focus on Endothelium-Derived Vasoactive Mediators*; Biota Publishing, 2011.
(10) Choi, H. W.; Barakat, A. I. Modulation of ATP/ADP Concentration at the Endothelial Cell Surface by Flow: Effect of Cell Topography. *Ann. Biomed. Eng.* **2009**, *37* (12), 2459–2468. https://doi.org/10.1007/s10439-009-9793-z.
